# Sibling species differently distributed around a CO_2_ vent show transplantation proteomic remodelling, while displaying metabolomic signatures associated with their origin

**DOI:** 10.1038/s41598-025-18913-y

**Published:** 2025-09-29

**Authors:** Lucy M. Turner, Diana Madeira, Elena Ricevuto, Alexia Massa Gallucci, Ulf Sommer, Mark R. Viant, Ramadoss Dineshram, Maria-Cristina Gambi, Piero Calosi

**Affiliations:** 1 Marine Biology & Ecology Research Centre, School of Biological and Marine Sciences, University of Plymouth, Plymouth, Devon, UK; 2 ECOMARE-Laboratory for Innovation and Sustainability of Marine Biological Resources, CESAM-Centre for Environmental and Marine Studies, Department of Biology, University of Aveiro, Gafanha da Nazaré, Portugal; 3 National Institute of Marine Biology, Ecology and Biotechnology, Stazione Zoologica Anton Dohrn, Department of Integrative Marine Ecology, Ischia Marine Centre, Ischia, Naples, Italy; 4 Istituto d’Istruzione Superiore Carlo Livi di Prato, Prato, Italy; 5 Blue EcoTech Ltd., Triq L-Indipendenza, Zebbug, Malta; 6 NERC Biomolecular Analysis Facility-Metabolomics Node (NBAF-B), University of Birmingham, Birmingham, UK; 7 School of Biosciences, University of Birmingham, Birmingham, UK; 8 The Swire Institute of Marine Sciences and School of Biological Sciences, The University of Hong Kong, Hong Kong, China; 9 Biological Oceanography Division, Council of Scientific and Industrial Research (CSIR), National Institute of Oceanography, Dona Paula, Goa India; 10 National Institute of Oceanography and Applied Geophysics, OGS, Trieste, Italy; 11 Marine Ecological and Evolutionary Physiology Laboratory, Département de Biologie Chimie et Géographie, Université du Québec à Rimouski, Rimouski, QC Canada

**Keywords:** Ocean acidification, *Platynereis*, Proteomics, Metabolomics, Lipidomics, Cellular stress response (CSR), Cell biology, Ecology, Molecular biology, Physiology, Ecology

## Abstract

The cellular homeostatic response (CHR) and cellular stress response (CSR) work together to maintain homeostasis. Studying phylogenetically closely-related species inhabiting different environments can help investigate the interplay between the CHR and CSR. We conducted reciprocal in situ transplant experiments in a natural CO_2_ vent (Ischia, Italy), using the sibling annelid species *Platynereis cf.*. *dumerilii* and *Platynereis cf.*. *massiliensis* which have been shown to have different preferential distributions around the CO_2_ vent. Following transplantations, we characterised the response of each individual’s proteome, metabolome, and lipidome, to short or long-term exposure to different *p*CO_2_ regimes (i.e., high and low), and confirmed its genetic identity. Here we show that different components of the CHR and CSR are utilised at different rates when *Platynereis* spp. are exposed to different *p*CO_2_ regimes, with cellular responses shown to be conserved across species. Metabolome and lipidome responses were dependent on regime of origin, and changed relatively slowly, whereas proteome responses were dependent on transplant type and changed more rapidly. Our results provide new insights to improve our understanding of the interplay between different cellular physiological responses involved in defining the functional phenotype of marine species, and their ability to acclimatise to future projected high *p*CO_2_ conditions.

**Supplementary Information:**

The online version contains supplementary material available at 10.1038/s41598-025-18913-y.

## Introduction

The cellular homeostatic response (CHR) and cellular stress response (CSR) work together to maintain cells’ homeostasis^1^. The CHR maintains the homeostasis of physiological set-points. When stress severity exceeds the limits of cellular homeostasis, the CSR is activated. The CSR is essential to enable an organism to cope with sub-optimal conditions, removing excessive damage that can accumulate during such periods. Thus investigating the interplay between CHR and CSR can help shed light on species’ sensitivity to current and future environmental changes^2–6^. However, these types of cellular adjustments can be highly species-specific, with even phylogenetically closely-related species utilising different molecular pathways to cope with changes in environmental conditions, ultimately possessing different homeostatic abilities^7–10^.

One such set of changes projected for the marine environment is an increase in seawater *p*CO_2_, as a consequence of ongoing anthropogenic atmospheric CO_2_ emissions, which will result in a decrease in mean pH of 0.4–0.5 pH units for the global ocean by the year 2100 and a change in the seawater carbonate system: a process termed ocean acidification (OA)^11,12^. We already know that these changes in seawater chemistry can have significant detrimental effects on marine species, communities and ecosystems^4,13,14^. However, in the case of OA, the vast majority of studies conducted are laboratory based, thus lacking the ecological complexity of natural settings, which can help modulate species’ responses to high *p*CO_2_/low pH (hereafter high *p*CO_2_) conditions^15^. In this sense, shallow water high *p*CO_2_ vents represent useful analogues to fully investigate the potential ecological and evolutionary implications of OA by means of field experiments^16–19^. Furthermore, we now have a plethora of studies examining the extent of marine species’ physiological plasticity (here defined as the phenotypic plasticity of physiological traits) and adaptation (here defined as selection of genotypes associated to physiological phenotypes)^20^ to high *p*CO_2_ conditions, including on their acid-base capabilities^21,22^ metabolism^3,16,23–26^ reproduction^27–29^ immune system^30^ oxidative stress tolerance^31–33^ and gene expression^34–39^. This said, the molecular mechanisms that underpin the spectrum of species’ plastic ability as the CHR and CSR to cope with high *p*CO_2_ conditions, or indeed to other drivers of marine global change, such as warming are hugely variable^3,10,40^. These can depend upon life stage, ecological history, and other factors, and are broadly speaking not yet fully characterised in marine organisms^5,8,14,41–43^.

The use of proteomics and metabolomics (including lipidomics) has begun to provide new insights into the molecular physiological mechanisms that dictate the extent of the plasticity of the CHR and CSR of marine species, including fish^44–47^ molluscs^10,48–54^crustaceans^42,55,56^ and annelids^8,57^ in response to exposure to global change drivers^58^. Integrated multiple omics approaches can assist in understanding the interplay among different molecules, assess the flow of information from one omics level to the other, and thus help to bridge the gap in our understanding of the links existing between genotypes and functional phenotypes^59–61^.

Annelids of the genus *Platynereis* living around the high *p*CO_2_ vent on the island of Ischia (Italy), have been utilised in several recent studies to characterise molecular signatures associated with OA across ecologically relevant gradients, capturing the full complexity of the environment^16,24,27,31,32,62–64^. These have included studies on whole-organism metabolic rates^21^ antioxidant capacity^31^ gene expression underpinning acid-base balance^34,36^ and metabolic machinery^35,37^ antioxidant efficiency^65^ and reproduction^66^. Previously, based on genetic data and differences in the whole-organism metabolic rate response to high and low *p*CO_2_, there were considered to be two separate lineages of *P. dumerilii* present in the waters surrounding the island of Ischia^16^. However, more recent work has confirmed that these lineages are in fact two sibling species^37,63,66^. The first of these, *Platynereis cf.*. *dumerilii* is found preferentially outside the CO_2_ vent (10:1 with *P. dumerilii*^66^ and displays an extreme reduction in metabolic rates when exposed to high *p*CO_2_ conditions^16^. This species is also a broadcast spawner^63,66^. By contrast, the second is a brooding species^63,66^*Platynereis cf.*. *massiliensis* (Moquin-Tandon, 1869), and is found preferentially inside the CO_2_ vent (15:1 with *P. massiliensis*^66^, and is able to maintain higher metabolic rates under high *p*CO_2_ conditions^16^. This species system provides us with the unique opportunity to gain new insights into the mechanisms of cellular remodelling that are involved in defining marine species’ ability (or lack of) to acclimatise and adapt to high *p*CO_2_ conditions, such as those that will be found in the future high-CO_2_ ocean.

In the present study we use a multi-omics (proteomics, lipidomics, and metabolomics) approach to characterise the extent of molecular physiological adjustment underpinning the CHR and CSR that sibling species of the genus *Platynereis* utilise when they were exposed to a change in *p*CO_2_ conditions. We hypothesised that the CHR and CSR molecular response to low or high *p*CO_2_ conditions during transplant would differ in annelids long-term exposed in situ to low or high *p*CO_2_ regimes: i.e., that a significant interactive effect on the CHR and CSR exists between the shorter-term exposure during transplants and the longer-term (chronic) exposure. To address our hypothesis, we used an in situ reciprocal transplant experimental design where individuals from high or low *p*CO_2_ regimes were exposed (short-term) either to their *p*CO_2_ conditions of origin (high or low) or to a new *p*CO_2_ condition (low or high). This allowed us to (1) quantify the effect of exposure to high and low *p*CO_2_ conditions on components of the CHR and CSR, and thus help explain these two species’ different sensitivities to high *p*CO_2_ conditions, and (2) provide new insights into the mechanisms of cellular responses underpinning the CHR and CSR that are involved in defining marine species’ ability to acclimatise, and adapt to drivers of marine global changes.

## Results

Survival of *Platynereis* spp. under experimental conditions (Table S2) was broadly comparable to that reported for similar studies^31^ and no significant differences in survival were found between the treatment groups (ANOVA *F*_3,12_ = 0.742, *P* = 0.547).

### Phylogenetic analyses

COI sequences were obtained for 52 individuals from inside the vented area (high *p*CO_2_ *P. cf.*. *massiliensis*) and 34 individuals from outside the vented area (low *p*CO_2_ *P*. cf. *dumerilii*). Sequences were deposited in GenBank (Accession numbers ON964737-ON964822, Table S3). The total analysed alignment length used for phylogenetic analyses was 605 bp. The maximum likelihood tree (Figure S2) recovered two well-supported major clades (a + b). The first of these (a = bootstrap Support of 82%) contained a mixture of individuals (*P. cf.*. *massiliensis* and *P*. cf. *dumerilii*) collected from both outside (60%) and inside (40%) the vented area in a number of well-supported clades. The second major clade (b = bootstrap Support of 90%) contained individuals from inside the vented area only (*P. cf.*. *massiliensis*) as well as a single individual from outside the vented area.

To further explore the extent of genetic differentiation between annelids from both inside and outside the vented area, the sequences were collapsed into haplotypes (*n* = 18) (Figure S3). The most common haplotype (P9) overwhelmingly contained individuals collected from inside the vent (96.7%) and was identified as being most similar to the haplotype of the *P*. cf. *massiliensis* reference species^66^ (Table S5). A further two haplotypes were also identified as *P*. cf. *massiliensis* (P2 and P5), although these were dominated by individuals collected outside the vented area (Table S3). The second most common haplotype (P1) was identified as *P*. cf. *dumerilii* and contained individuals collected from both inside (45.8%) and outside (54.2%) the vented area (Table S3). Two more haplotypes were identified as *P*. cf. *dumerilii* (P3 and P15). Haplotype P3 contained a mix of individuals from inside and outside the vent, whereas P15 contained a single individual from inside the vent (Table S3). The remaining haplotypes contained only a single individual each and were unable to be assigned as either *P*. cf. *massiliensis* or *P*. cf. *dumerilii* and were therefore designated *Platynereis* sp. (Figure S3).

## Characterisation of metabolomics profiles

### Polar extracts

**Fig. 1:**
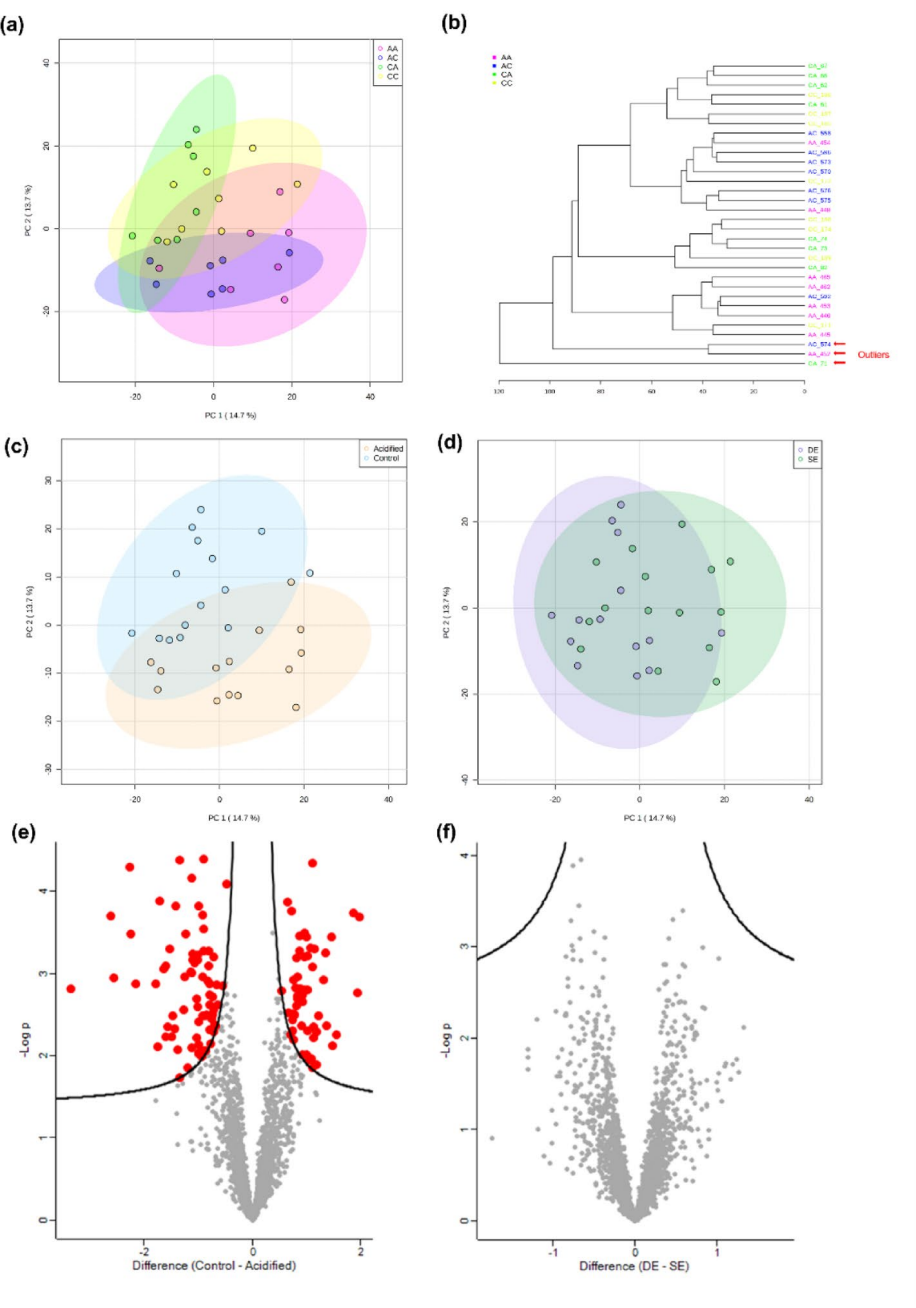
Metabolome (polar extracts) characterization of marine annelids *Platynereis* spp. reciprocally transplanted between high or low *p*CO_2_ conditions (four treatments, CC = collected in low *p*CO_2_ and transplanted to low *p*CO_2_, CA = collected in low *p*CO_2_ and transplanted to high *p*CO_2_, AA = collected in high *p*CO_2_ and transplanted to high *p*CO_2_, and AC = collected in high *p*CO_2_ and transplanted to low *p*CO_2_). **(a)** Principal Components Analysis 2D score plot of the four groups with 95% confidence intervals, **(b)** Sample cluster analysis using Euclidean distance and Ward’s clustering algorithm, **(c)** Principal Components Analysis 2D score plot of the metabolome of annelids according to regime of origin (control, including group CC and CA; and acidified, including group AA and AC) with 95% confidence intervals, **(d)** Principal Components Analysis 2D score plot of the metabolome of annelids according to transplant type, namely between the same environment (SE, which includes CC and AA annelids), or different environment (DE, which includes CA and AC annelids) with 95% confidence intervals. **(e)** Volcano plot representing differentially abundant metabolites (*n* = 129, coloured as red dots) according to worm regime of origin (control vs. acidified), based on *t*-tests with 250 randomizations, FDR 0.05 and s 0.1. **(f)** Volcano plot highlighting that there are no differences in the metabolome of annelids according to transplant type, based on *t*-tests with 250 randomizations, FDR 0.05 and s 0.1.

**Fig. 2:**
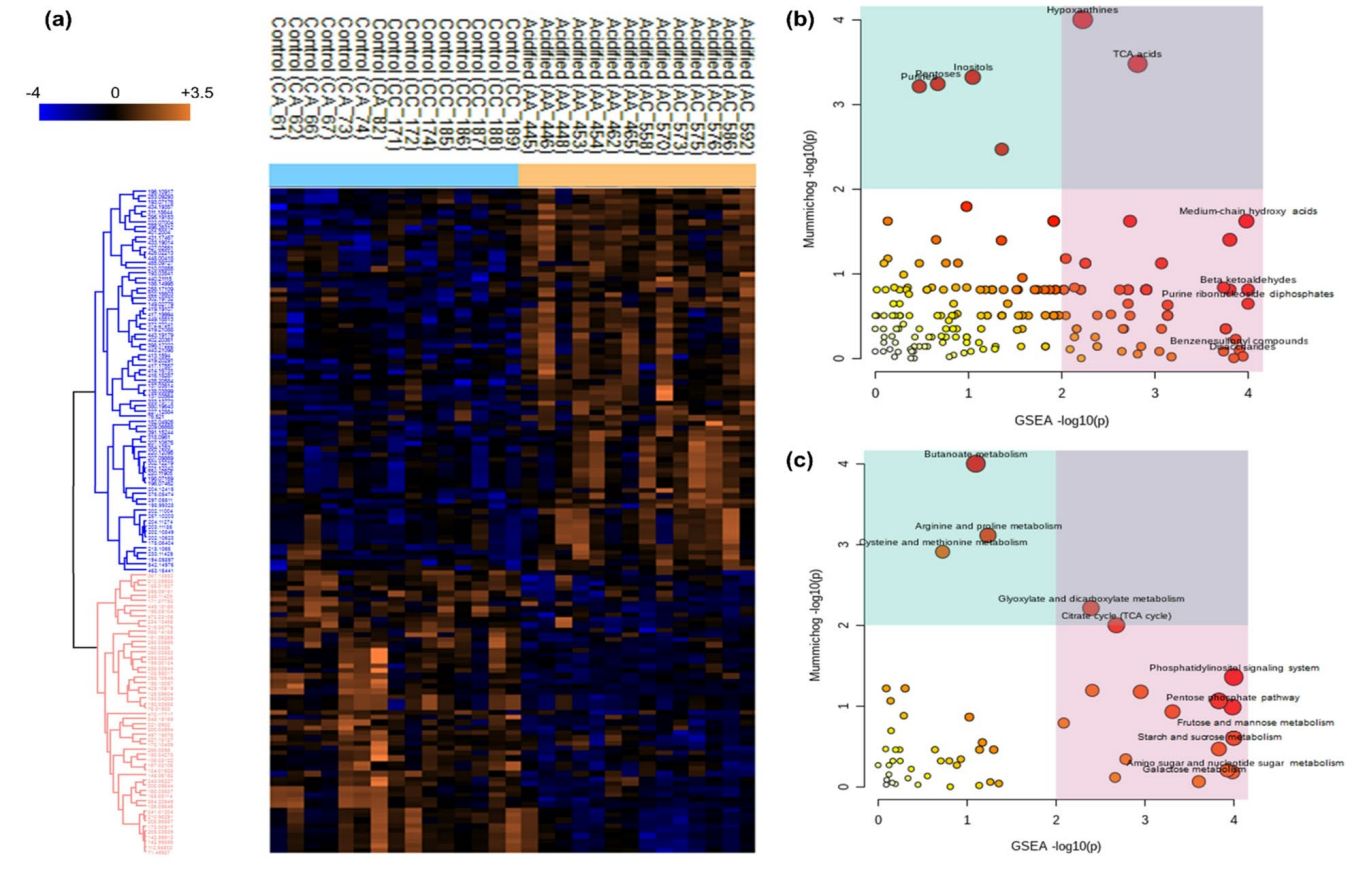
Metabolome (polar extracts) profiling of marine annelids *Platynereis* spp. according to regime of origin, namely control (including CC annelids = collected in low *p*CO_2_ and transplanted to low *p*CO_2_, and CA = collected in low *p*CO_2_ and transplanted to high *p*CO_2_) and acidified (including AA annelids = collected in high *p*CO_2_ and transplanted to high *p*CO_2_, and AC = collected in high *p*CO_2_ and transplanted to low *p*CO_2_). **(a)** Heat map representation of the 129 significantly different *m/z* peaks, based on a clustered data matrix (distance: Spearman correlation; linkage: complete) in which cells denote the normalized metabolite abundances (glog transformed and Z-scored), ranging from down-accumulated (blue) to up-accumulated (orange). **(b)** Top enriched metabolite classes based on mummichog and GSEA algorithms. **(c)** Top enriched pathways based on mummichog and GSEA algorithms. Graphs are coloured according to significance in mummichog (blue) and significance in GSEA (pink). The size and colour of the circles correspond to their transformed combined p-values. More details on enrichment results can be consulted on Table S4 and S5.

The metabolomics analysis of *Platynerei*s spp. led to a data matrix of 2,230 signals for polar extracts. The PCA showed some separation between groups (*p* = 0.0002; Fig. 1A, B,C, Table S4), with some degree of separation between CC (transplanted from low to low *p*CO_2_ conditions) and CA (transplanted from low to high *p*CO_2_ conditions) (*p* = 0.09), and between AA (transplanted from high to high *p*CO_2_ conditions) and AC (transplanted from high to low *p*CO_2_ conditions) (*p* = 0.05). In addition, a certain degree of separation was also observed between C-origin groups (CC and CA together) with A-origin groups (AA and AC together), suggesting a differentiation by regime of origin (*p* = 0.0001) (Fig. 1C, Table S4). The cumulative explained variance for the first two components was low: 28.4%. The volcano plot comparing regime of origin highlighted 129 differentially abundant *m/z* peaks (i.e., Differentially Abundant Metabolites (DAM)) between control and acidified sites (Fig. 1E, Table S6), and there were also differences found according to transplant type (*p* = 0.0303, Fig. 1D, F, Table S4), same environment vs. different environment). Among the 129 significant metabolites, 74 showed higher abundance and 55 showed lower abundance in specimens from an acidified environment, when compared to control (Fig. 2A). Among the DAM, 64 *m/z* peaks did not have an annotation (49.6%). According to the combination of mummichog and GSEA algorithms, enriched compound classes (combined *p* < 0.05) were hypoxanthines, TCA acids, inositols, medium-chain hydroxy acids, pentoses, purines, amino acid amides, pterins, indolizidines, beta ketoaldehydes, thioaldehyde s-oxides, pyrimidine ribonucleosides, purine ribonucleoside diphosphates, biopterins and diarylethers (Fig. 2B, Table S7). Other classes with a combined *p* > 0.05 but significant in the analysis of one of the algorithms include beta hydroxy acids (mummichog *p* < 0.05) and allyl-type 1,3-dipolar organic compounds, aryl-aldehydes, tricarboxylic acids, monosaccharide phosphates, cyclohexane Dicarboxylic acids, quinolidines, benzenesulfonyl compounds, Dissacharides, purine deoxyribonucleosides, benzoic acid esters, N-arylamides, metoxyphenols, and 1,2-diols (GSEA *p* < 0.05) (Fig. 2B, Table S7). The metabolic pathways significantly enriched in the dataset according to mummichog and GSEA combined *p* < 0.05 include butanoate metabolism; phosphatidylinositol signaling system; pentose phosphate pathway; phenylalanine, tyrosine and tryptophan biosynthesis; ubiquinone and other terpenoid-quinone biosynthesis; Citrate (TCA) cycle; glyoxylate and dicarboxylate metabolism; arginine and proline metabolism; fructose and mannose metabolism; starch and sucrose metabolism (Fig. 2C, Table S8). Other pathways with a combined *p* > 0.05 but significant in the analysis of one of the algorithms include cysteine and methionine metabolism (mummichog *p* < 0.05), and glycolysis/gluconeogenesis, phenylalanine metabolism, inositol phosphate metabolism, amino sugar and nucleotide sugar metabolism, galactose metabolism and caffeine metabolism (GSEA *p* < 0.05) (Fig. 2C, Table S8).

### Lipid extracts

**Fig. 3:**
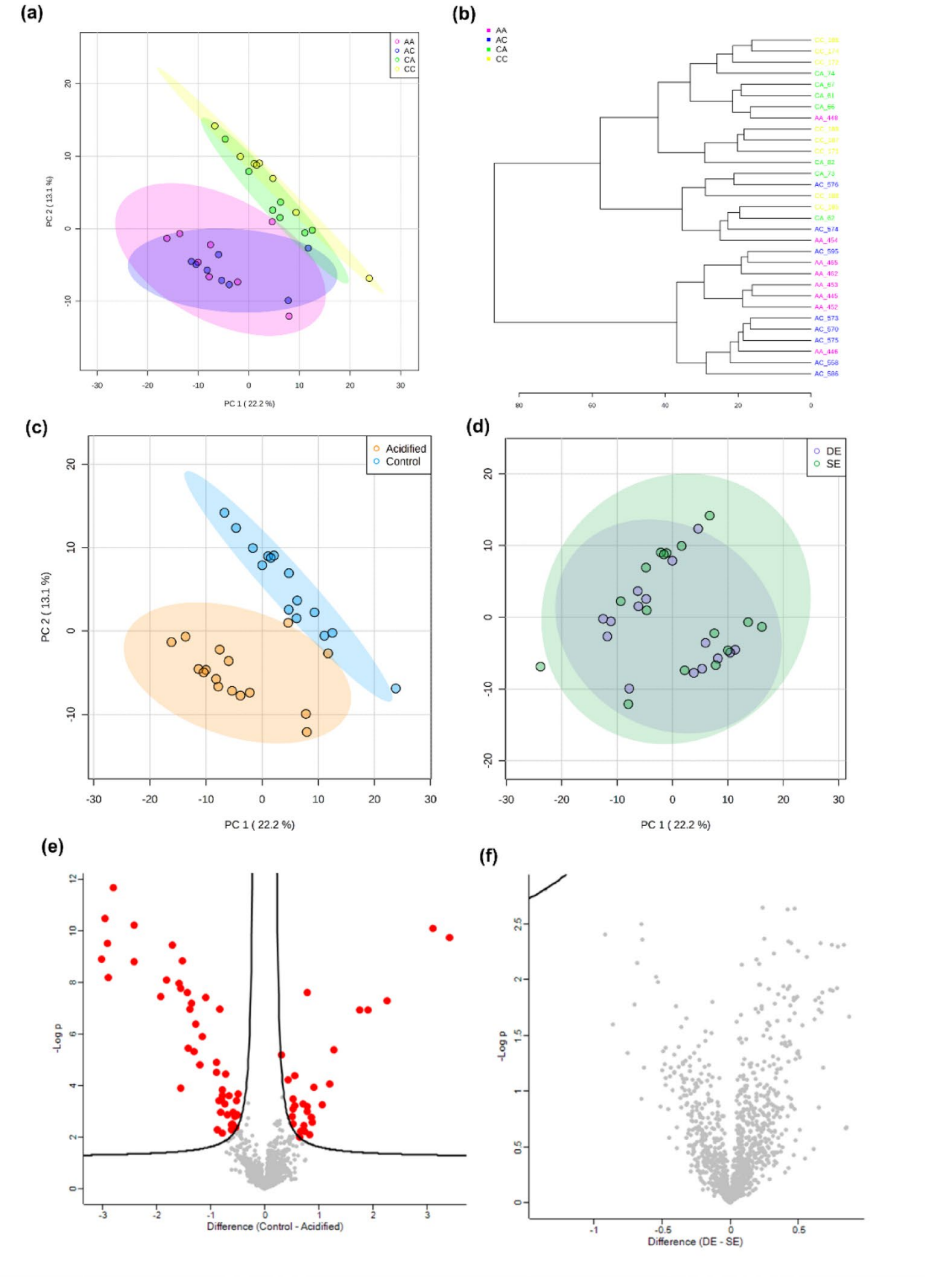
Lipidome characterization of marine annelids *Platynereis* spp. reciprocally transplanted between high or low CO_2_ conditions (four treatments, CC = collected in low *p*CO_2_ and transplanted to low *p*CO_2_, CA = collected in low *p*CO_2_ and transplanted to high *p*CO_2_, AA = collected in high *p*CO_2_ and transplanted to high *p*CO_2_, and AC = collected in high *p*CO_2_ and transplanted to low *p*CO_2_). **(a)** Principal Components Analysis 2D score plot of the four groups with 95% confidence intervals, **(b)** Sample cluster analysis using Euclidean distance and Ward’s clustering algorithm, **(c)** Principal Components Analysis 2D score plot of the lipidome of annelids according to regime of origin (control, including group CC and CA; and acidified, including group AA and AC) with 95% confidence intervals, **(d)** Principal Components Analysis 2D score plot of the lipidome of annelids according to transplant type, namely between the same environment (SE, which includes CC and AA annelids), or different environment (DE, which includes CA and AC annelids) with 95% confidence intervals. **(e)** Volcano plot representing differentially abundant lipids (coloured as red dots) according to annelid regime of origin (control vs. acidified), based on *t*-tests with 250 randomizations, FDR 0.05 and s 0.1. **(f)** Volcano plot highlighting that there are no differences in the lipidome of annelids according to transplant type, based on *t*-tests with 250 randomizations, FDR 0.05 and s 0.1.

**Fig. 4:**
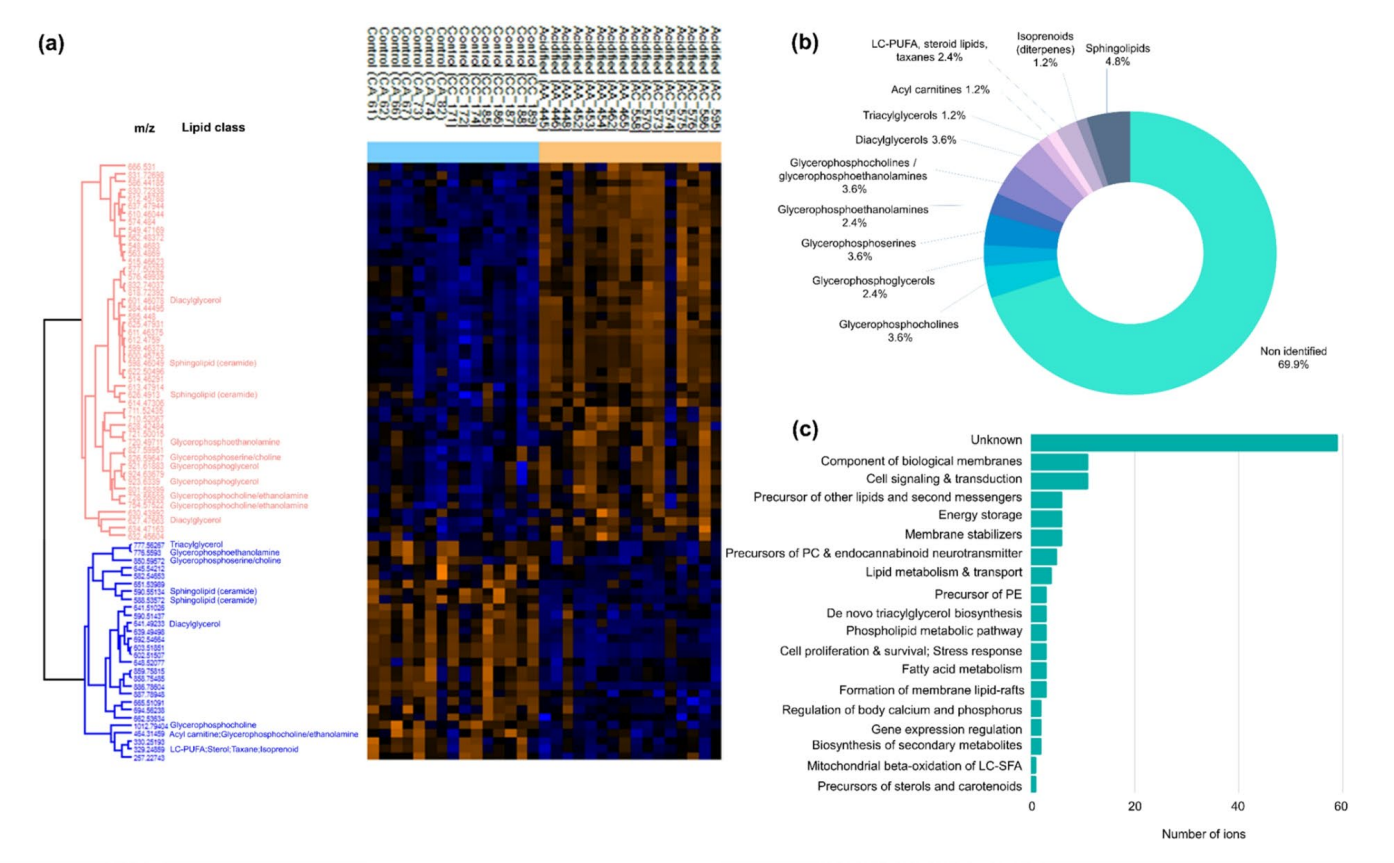
Lipidome profiling of marine annelids *Platynereis* spp. according to regime of origin, namely control (including CC annelids = collected in low *p*CO_2_ and transplanted to low *p*CO_2_, and CA = collected in low *p*CO_2_ and transplanted to high *p*CO_2_) and acidified (including AA annelids = collected in high *p*CO_2_ and transplanted to high *p*CO_2_, and AC = collected in high *p*CO_2_ and transplanted to low *p*CO_2_). **(a)** Heat map representation of the clustered data matrix (distance: Spearman correlation; linkage: complete) in which cells denote the normalized lipid abundances (glog transformed and Z-scored), ranging from down-accumulated (blue) to up-accumulated (orange). **(b)** Main lipid classes among the significant *m/z* peaks and corresponding ions. **(c)** Biological functions (retrieved from the Encyclopedia of Lipidomics and HMDB Metabocards) associated with the lipid classes of the identified ions (each ion may be associated to several biological functions). PC – glycerophosphocholines, PE – glycerophosphoethanolamines, LC-SFA – long chain saturated fatty acids.

The lipidomics analysis of *Platynereis* spp. led to a data matrix of 1,206 signals for lipid extracts. The PCA showed an overlap between groups CC and CA, which separated from groups AA and AC, confirming a separation by regime of origin (*p* = 0.0001, Fig. 3C, Table S4), also observable in the sample cluster analysis (Fig. 3A, B, C). The cumulative explained variance for the first two components was 35.3%. The volcano plot comparing regime of origin highlighted 76 Differentially Abundant Lipids (DAL) between control and acidified sites (Fig. 3E, Table S8) whereas no differences were found according to transplant type (*p* = 0.2391) (Fig. 3D, F, Table S4, same environment vs. different environment). Among the 76 significant lipids, 48 showed higher abundance and 28 showed lower abundance in annelids from an acidified environment, when compared to control (Fig. 4A). Most of the DAL did not have an annotation (69.9%) and those that were putatively annotated belong to the main lipid classes of sphingolipids, isoprenoids, acyl carnitines, triacylglycerols, diacylglycerols, glycerophosphocholines, glycerophosphoserines, glycerophosphoethanolamines, glycerophosphoglycerols and LC-PUFA/steroid lipids/taxanes (Fig. 4B). The biological functions associated to these lipid classes include component of biological membranes, cell signalling & transduction, precursor of other lipids and second messengers, energy storage, membrane stabilizers, de novo triacylglycerol biosynthesis, fatty acid metabolism, cell survival and stress response, regulation of body calcium and phosphorus, gene expression regulation and precursors of sterols and carotenoids (Fig. 4C, Table S9). The enrichment analysis showed that eight lipid classes were enriched according to GSEA algorithm, namely glycerophosphoserines, glycerophosphoinositols, diradyglycerols, glycosyldiradylglycerols, fatty esters, ceramides, fatty acids, sphingomyelins (Table S10). The mummichog algorithm did not produce any significantly enriched classes, resulting in combined *p* > 0.05.

### Protein profiling

**Fig. 5:**
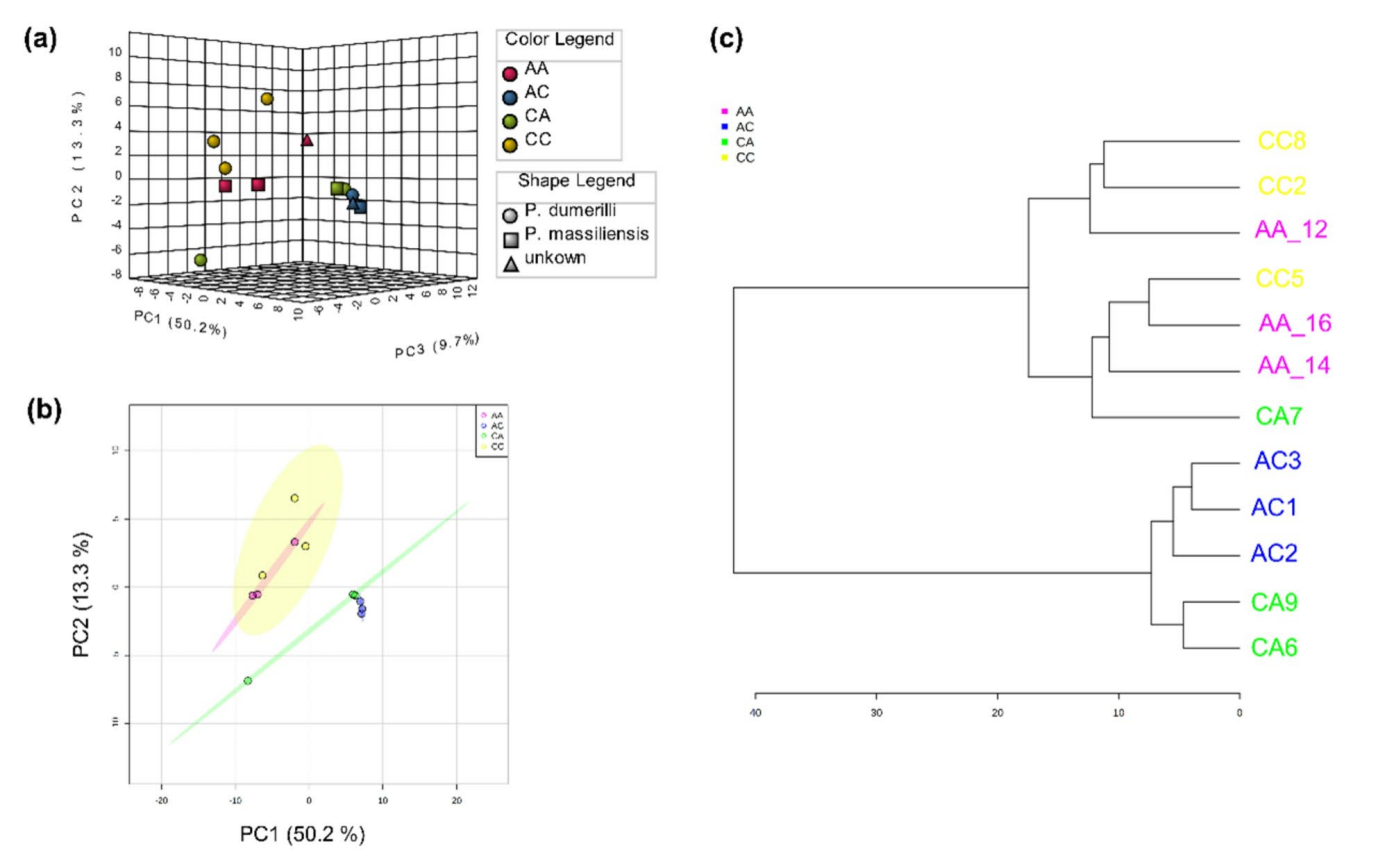
Proteome characterization of marine annelids *Platynereis* spp. reciprocally transplanted between high or low CO_2_ conditions (four treatments, CC = collected in low *p*CO_2_ and transplanted to low *p*CO_2_, CA = collected in low *p*CO_2_ and transplanted to high *p*CO_2_, AA = collected in high *p*CO_2_ and transplanted to high *p*CO_2_, and AC = collected in high *p*CO_2_ and transplanted to low *p*CO_2_). Species were identified (using COI) as *Platynereis cf.*. *dumerilii*, *Platynereis cf.*. *massiliensis* or unknown. **(a)** Principal Components Analysis 3D score plot, **(b)** Principal Components Analysis 2D score plot with 95% confidence intervals **(c)** Sample cluster analysis using Euclidean distance and Ward’s clustering algorithm.

**Fig. 6:**
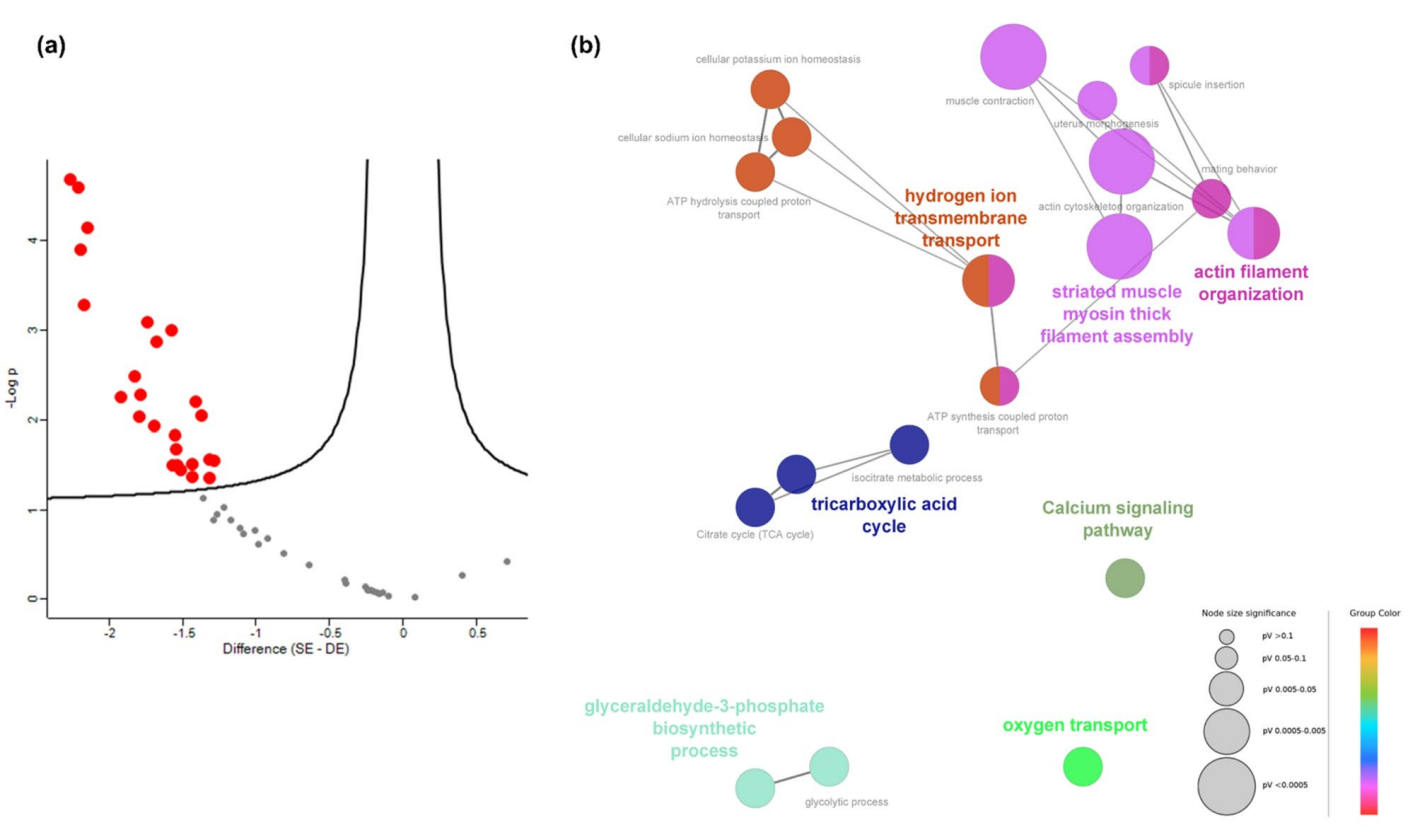
Differentially abundant proteins (DAPs) and pathways regulated by marine annelids *Platynereis* spp. reciprocally transplanted between the same (SE) or different environments (DE), namely high or low *p*CO_2_ conditions. The group SE includes CC annelids (collected in low *p*CO_2_ and transplanted to low *p*CO_2_) and AA annelids (collected in high *p*CO_2_ and transplanted to high *p*CO_2_). The group DE includes CA annelids (collected in low *p*CO_2_ and transplanted to high *p*CO_2_) and AC annelids (collected in high *p*CO_2_ and transplanted to low *p*CO_2_). **(a)** Volcano plot representing the proteome difference between annelids transplanted to the SE and DE, based on *t*-tests with 250 randomizations, FDR 0.05 and s 0.1. **(b)** Network analysis carried out using Cytoscape v3.8.0 and plugins ClueGO v2.5.7 and CluePedia v1.5.7. The analysis was based on differentially abundant proteins (DAPs) between SE and DE (species: *Caenorhabditis elegans*, ontologies: GO_ImmuneSystemProcess-GOA_17.11.2016_10h53, KEGG_17.11.2016, GO_BiologicalProcess-GOA_17.11.2016_10h53, enrichment/depletion: two-sided hypergeometric with Bonferroni step down correction and p-value of 0.05, GO level 3 to 8, minimum number of genes 1, minimum percentage of genes 4%, Kappa score 0.4).

**Fig. 7:**
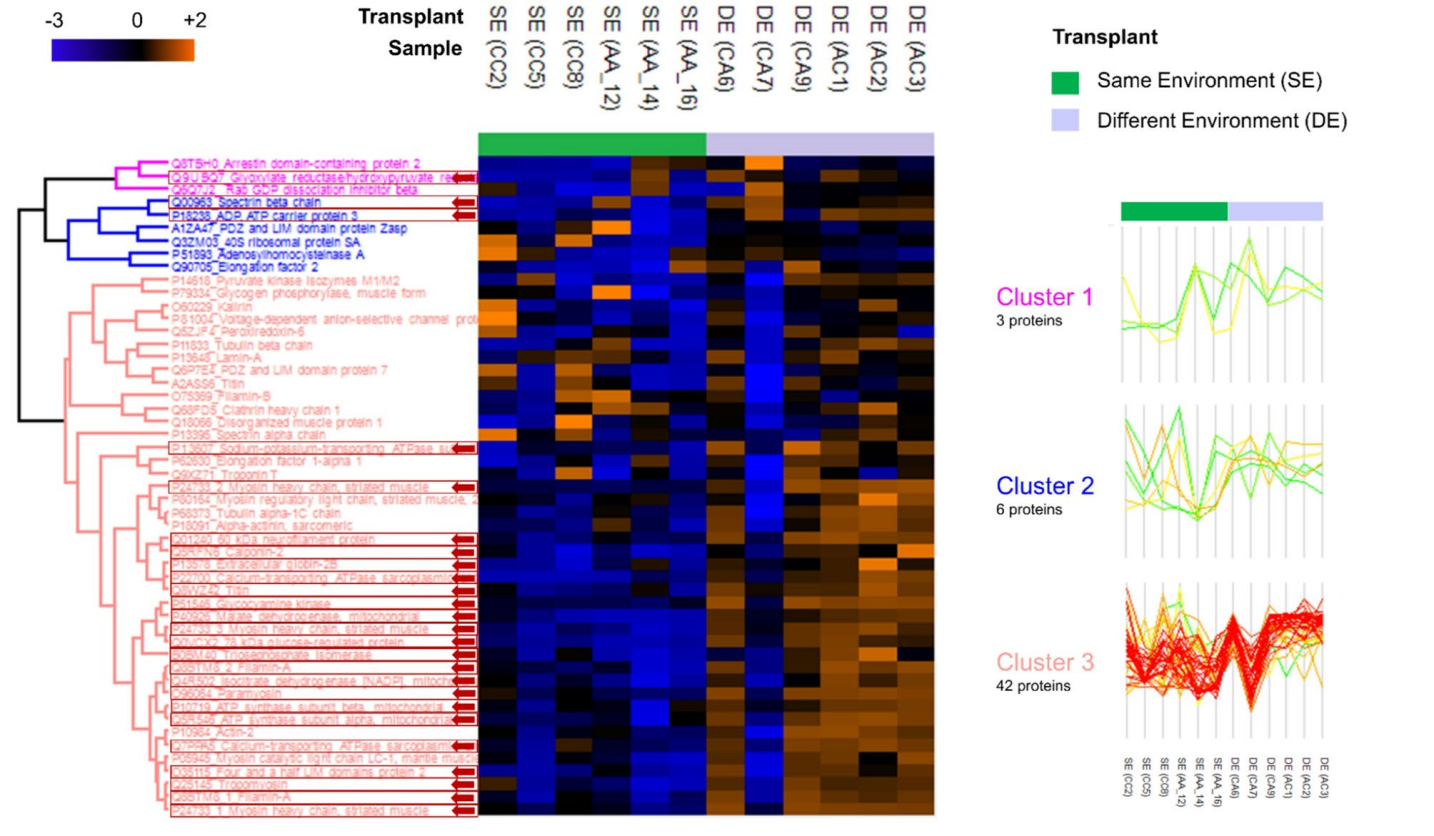
Proteomic profiling of marine annelids *Platynereis* spp. reciprocally transplanted between the same environment (SE) or different environment (DE), namely high or low CO_2_ conditions (four treatments, CC = collected in low *p*CO_2_ and transplanted to low *p*CO_2_, CA = collected in low *p*CO_2_ and transplanted to high *p*CO_2_, AA = collected in high *p*CO_2_ and transplanted to high *p*CO_2_, and AC = collected in high *p*CO_2_ and transplanted to low *p*CO_2_). Heat map representation of the clustered data matrix (distance: Spearman correlation; linkage: complete) in which cells denote the normalized protein abundances (Log_2_ transformed and Z-scored), ranging from down-accumulated (blue) to up-accumulated (orange). Differentially abundant proteins are highlighted with an arrow (based on a volcano plot - *t*-tests with 250 randomizations, FDR 0.05 and s 0.1). Three clusters were defined, and protein profiles were plotted for each cluster.

**Fig. 8:**
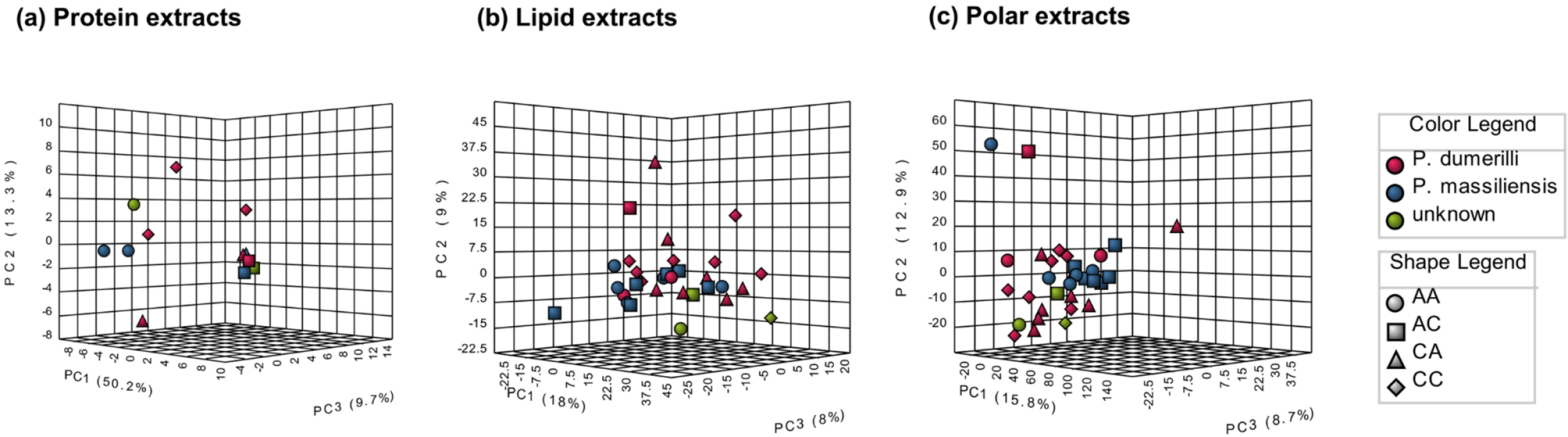
Multi-omics characterization of marine annelids *Platynereis* spp. reciprocally transplanted between high or low CO_2_ conditions (four treatments, CC = collected in low *p*CO_2_ and transplanted to low *p*CO_2_, CA = collected in low *p*CO_2_ and transplanted to high *p*CO_2_, AA = collected in high *p*CO_2_ and transplanted to high *p*CO_2_, and AC = collected in high *p*CO_2_ and transplanted to low *p*CO_2_). Species were identified (using COI) as *Platynereis cf.*. *dumerilii*,* Platynereis cf.*. *massiliensis* or unknown. Principal Components Analysis score plot of the (a) proteome, (b) metabolome, namely lipid extracts (c) metabolome, namely polar extracts.

The proteomics analysis of *Platynereis* spp. led to the identification of 663 proteins, 51 of which remained for statistical analysis after the filtering step (removal of proteins with < 60% valid values). The PCA showed an overlap between groups CC and AA, which separated from groups AC and CA (*p* = 0.0277, Fig. 5A, B, Table S4), with this pattern being confirmed by the sample cluster analysis (Fig. 5C). With regards to species, no clusters were evident in the PCA. The cumulative explained variance for the first two components was 63.5%. While there were no differences in the annelids’ proteome according to regime of origin (*p* = 0.628), the volcano plot highlighted 25 proteins that significantly changed (Differentially Abundant Proteins (DAPs)) between transplant type (*p* = 0.0118), having higher abundance in the Different Environment (DE) group when compared to the Same Environment (SE) group (Fig. 6A, Table S11). Proteome variation across samples was divided into three clusters (Fig. 7). Proteins in cluster 1 seem to show a slight trend of increasing abundance from SE to DE, whereas proteins in cluster 2 show no Discernible abundance pattern. However, there seems to be a reduction in variance from SE to DE. Finally, cluster 3 proteins show a trend of higher abundance towards DE, when compared to SE (Fig. 7). Based on DAPs, 19 gene ontology (GO) terms were significantly enriched in the network analysis, the most relevant being hydrogen ion transmembrane transport, actin filament organisation, striated muscle myosin thick filament assembly, tricarboxylic acid cycle, calcium signalling pathway, oxygen transport and glyceraldehyde-3-phosphate biosynthetic process (Fig. 6B). Multi-omics analysis of combined metabolomics and proteomics data (Fig. 8) confirmed that there is no evident separation of responses by species.

## Discussion

We show that different molecular components (i.e., proteome, lipidome, and metabolome) of the CHR and CSR are utilised at different rates when *Platynereis* spp. are exposed in situ to different *p*CO_2_ conditions. Using a multi-omics approach has enabled us to demonstrate that cellular responses at the level of the metabolome and lipidome largely depend on the regime of origin (i.e., long-term chronic exposure to low or high *p*CO_2_), and therefore change relatively slowly, whereas responses at the level of the proteome depend on transplant type (i.e., short-term acute exposure to low or high *p*CO_2_) and change relatively quickly by comparison. Our finding that these relatively fast proteomic changes were directly related to short-term acute exposure (transplant type) correlates with what we might expect from these being representative of the direct consequences of transcription and protein turnover^67,68^. By comparison, the slower rate of metabolomic changes attributable to long-term chronic exposure (regime of origin) can be matched with the fact that these are representative of the relatively slower downstream metabolic consequences of these changes and the regulatory feedback mechanisms that act on these^69,70^. However, our results also demonstrate that differences in the rates of molecular responses in different omics compartments remain highly conserved in phylogenetically closely-related species that inhabit different *p*CO_2_ regimes.

Our results differed from what we had hypothesised based on the different spatial distribution, as well as previous genetic and whole-organism physiological evidence, of the sibling species investigated. Our results demonstrate that despite showing a different preferential distribution around the CO_2_ vent, and whole-organism physiology and life-history adaptions to different *p*CO_2_ regimes^16,66^ cell physiology responses that are part of the CSR of these two sibling species are in fact comparable, and highly conserved when individuals are transplanted to a new *p*CO_2_ environment. These results suggest that, upon transfer to stressful conditions, here a different *p*CO_2_ condition, from that of their origin, *Platynereis* spp. may activate a set of conserved cellular pathways as part of the CSR to ensure survival^71,72^. Our use of a multi-omics approach provides new insights into the rates of deployment of the different mechanisms implicated with the CHR and CSR that are involved in defining marine species’ ability to cope with OA found in the natural CO_2_ vents. We develop our discussion around *Platynereis* spp. adaptive and plastic responses of fundamental cell functions to different *p*CO_2_ in situ.

### Membrane transport and cell signalling

Cell membranes can be considered the single most important feature of any cell and the key component of the CHR. They control the flow of ions, both passively and actively, in and out, maintaining internal cell pH and facilitating cell signalling. These two processes together ensure the fundamental maintenance of cellular homeostasis^73^. Here we report an increase in the abundance of proteins involved in hydrogen ion transmembrane transport in individuals that were transplanted to a new *p*CO_2_ condition. This is suggestive of adjustments to cellular acid-base balance upon exposure to a new *p*CO_2_ condition, *via* buffering and active ion transport processes, which would prevent strong changes in cell acid–base chemistry. Marine invertebrates such as crustaceans and echinoderms that inhabit coastal environments, thus experiencing frequent fluctuations in *p*CO_2_, are known to possess an elevated capacity to compensate for acute extracellular acid–base disturbances *via* ion transport proteins and other osmo-ionic regulation mechanisms^74,75^. Marine species were also shown to upregulate these mechanisms when exposed to high *p*CO_2_^4^. It is therefore not surprising that in our study both species, when transplanted to a new (and differing) *p*CO_2_ environment, respond similarly by upregulating their acid-base buffering capabilities *via* membrane ion exchange. This points to the fact that this component of the CHR is possibly highly conserved, as it does not change with acute changes in *p*CO_2_ conditions.

At the same time, the lipidome of individuals differs according to the regime of origin. However, by contrast to changes at the level of the proteome which mainly highlighted changes in proteins involved in hydrogen ion transmembrane transport as an important component of the CHR, annelids from high rather than low *p*CO_2_ regimes (regardless of species) showed higher concentrations of lipids associated with cell membranes (and lipid rafts), cell signalling and transduction, including precursors of other lipids and second messengers. Repair and prevention of macromolecular damage (including to cell and organelle membranes) and activation of cell cycle checkpoints are two of the cornerstones of the CSR^72^. Our results support this and suggest that cell membrane adjustments and associated cell signalling pathways are important features of the CSR, but also the CHR in long-term acclimatisation/adaptation to high *p*CO_2_ conditions. However, we cannot exclude that the patterns observed are (at least in part) determined by ecological conditions of habitat and food availability that we know can differ inside and outside the CO_2_ vent^62,76^.

On the other hand, we know that for other homeostatic mechanisms, such as increased ion and acid-base regulation under high *p*CO_2_ conditions, there is an increase in metabolic costs, often accompanied by the reallocation of energy budgets away from growth and towards homeostasis^6^. Indeed mobilisation and reallocation of metabolic energy is a cornerstone of the CSR^72^. In agreement with this idea, individuals of *P. massiliensis*, which dominate inside the Ischia CO_2_ vents, were shown to possess higher metabolic rates^16^ and display a smaller body size^16,66^.

### Metabolism

In general, when compared to annelids that are adapted to the low *p*CO_2_ regime those adapted to the high *p*CO_2_ regime show increased concentrations of metabolites, and an upregulation of pathways that are predominantly related to key CSR components of energy metabolism, including the pentose-phosphate pathway, the citrate cycle, glycolysis, fatty acid metabolism, and amino acid, nucleotide and carbohydrate production^72^. Changes in concentrations of metabolites involved in energy metabolism were also detected in the mussel *Mytilus galloprovincialis* that inhabits the same high *p*CO_2_ vent system, when compared to those that inhabit low *p*CO_2_ areas^77^. However, in our study these differences persist in annelids following exposure to a new *p*CO_2_ condition as is the case for the scallops, *Pecten maximus*^78^ and *Argopecten irradians irradians*^79^ as well as the copepod *Calanus glacialis*^80^ when exposed to high *p*CO_2_. The enhancement of major pathways such as glycolysis, citrate cycle and fatty acid metabolism in annelids, and mussels from the high *p*CO_2_ regime may be an adaptation to increase energy production in energy-demanding environmental conditions to support the documented increase in the cost of life they face^16,77^. Amino acid upregulation was also recorded for the shrimp *Pandalus borealis* exposed to high *p*CO_2_ conditions^81^. Amino acid regulation may also be relevant in buffering high-energy phosphate levels in invertebrates exposed to high *p*CO_2_ conditions. Moreover, several amino acids may serve as organic osmolytes for osmotic control, the importance of which has been demonstrated to be central in enabling marine invertebrates including the crab *Carcinus maenas* and lobster *Homarus americanus* to cope with OA conditions^42,82^. Thus, the regime of origin appears important in determining baseline levels of energy production pathways in *Platynereis* spp.

On the contrary, whereas changes to the proteome were also associated with energy metabolism, these related to the short-term transplantation to a new environment, and not to the regime of origin. This suggests that a CSR is likely induced in individuals having to deal with the stress imposed by exposure to new environmental conditions^71^. Compared to the effects of exposure to changes in *p*CO_2_ much of the previous work on marine invertebrates has been conducted characterising the effects of temperature on the CSR^71^. However, whilst previous work characterising the response of marine metazoans exposed to high *p*CO_2_ conditions has often reported an increase in energy metabolism^6^ including at the level of the proteome in marine invertebrates such as the oyster *Crassostrea gigas*, lobster *Homarus americanus*, and crab *Cancer magister*^42,49,52,56,83,84^ by contrast other studies have shown metabolic downregulation or depression^16,50,85^. We report differences in the underlying molecular (metabolomic and lipidomic) physiological machinery in closely related species adapted to different levels of *p*CO_2_. Thus our results agree with the notion that responses to high *p*CO_2_ levels are highly species specific^86^.

### Cytoskeletal proteins and cell survival

The cytoskeleton is a network of protein filaments, including actin and myosin that extends from the cell nucleus to the cell membrane, anchoring the internal structural components and providing structural stability to the cell^73^. It also plays an important role in cell signalling^87^ a fundamental component of the control of the CSR^72^. We report an increased abundance of proteins involved in actin filament organisation and striated muscle myosin thick filament assembly in annelids transplanted to new *p*CO_2_ conditions. Exposure to high *p*CO_2_ conditions can induce a generalised stress response (e.g., CSR) through changes in the electron transport chain and generation of reactive oxygen species that in turn can cause oxidative stress^88^. This can then lead to cellular damage and disruption of the cytoskeleton including the actin and tubulin complex, which is then compensated by upregulation of cytoskeletal proteins, as observed in the South African abalone *Haliotis midae*^53^. Cytoskeletal adjustments have also been recorded in fish^44^ molluscs^89^ and crustaceans^90^ suggesting that this response is conserved across metazoans as the cytoskeleton is a major target of stress and stress-induced ROS.

In our study the upregulation in these proteins occurs regardless of the level of *p*CO_2_ that annelids were transplanted toward. However, the lipidome of annelids from the high *p*CO_2_ regime undergoes an increase in the concentration of lipids associated with cell survival and the CSR, as well as oxygen transport, suggesting that extant adaptation to high *p*CO_2_ can enable this suite of responses to facilitate survival under hypercapnic conditions. Whereas previous work in marine invertebrates such as *C. gigas* and *H. midae* has demonstrated an increase in proteins related to the CSR^49,53^ following exposure to high *p*CO_2_ conditions, other studies have recorded either an upregulation in the same species^53,88^ or a suppression of those proteins mapped to the cytoskeleton scaffold in other marine bivalve species such as *C. gigas* and the clam *Panopea generosa*^49,54^.

## Conclusions

Global changes are projected to negatively affect marine biota and ecosystems. In order to understand the capacity of marine organisms to acclimatise and adapt to these changes, it is vital we gain a thorough understanding of the physiological mechanisms they are able to deploy in the attempt to cope with changing environmental conditions^91–93^. Integrating a multi-omics approach with in situ transplant experiments conducted on two sibling species with a documented differential distribution in relation to different seawater *p*CO_2_ conditions, has been fundamental in allowing us to gain new insights into the rate of deployment of molecular level mechanisms involved that dictate the CHR and CSR responses that underpin organisms’ acclimatisation and adaptation to OA conditions.

Our results allow us to conclude that upon transplantation (i.e., transfer to a new *p*CO_2_ condition), organisms may activate a set of conserved pathways which are part of the CHR and CSR to enhance survival, while regime of origin (i.e., previous environmental history/adaptation) may determine the baseline levels or thresholds for molecules and cellular pathways as part of the CHR and CSR. This is somewhat in contrast with the results previously reported^8^ from two phylogenetically-closely related species of marine annelids of the genus *Ophryotrocha* which possess greatly different biogeographies (i.e., rare vs. common species) and showed distinctive metabolomics responses when exposed to ocean warming conditions, both alone and combined with OA. Research aiming at understanding the cellular mechanisms underpinning the CHR and CSR to global change drivers of marine species, populations and communities is broadly speaking in its infancy. The use of an in situ reciprocal transplant experimental design including morphologically cryptic species such as we have deployed here remains logistically challenging. For example, we recognise there are differences in mortality rates between some treatment groups, not all sampled individuals were able to be identified to species, and that we only sampled at a single time point, meaning we were unable to draw any inferences about the temporal progression of CHR and CSR processes in different omics compartments. The identification of the omics level processes characterising the progression of CHR and CSR over time remains an avenue for further work. Also, we cannot completely exclude that the specific in situ transplantation conditions, whilst meticulously standardised, may have possibly partly influenced our results. To this end future studies may include the omic profiling and comparison of non-transplanted specimens to enhance our understanding of baseline responses before evaluating the detailed response of the CHR and CSR to differing CO₂ environments. However, in order to acquire a more in-depth understanding of the diversity of physiological and cellular mechanisms marine organisms will be able to rely on in the future high *p*CO_2_ ocean, we must continue to move beyond those relatively few well characterised taxa, and systems, to enable us to simultaneously shed light on the responses of individuals across different levels of biological organisation^61,94^. Ultimately, these new frontiers in global change biology will help us acquire a further critical understanding of the way biotic systems function, have evolved, and may further evolve, under rapid environmental changes.

## Materials and methods

### Specimen collection and Preparation for in-situ transplant

*Platynereis* spp. (*n* = 106, mean wet Weight 1.59 ± 0.02 mg) were sampled by means of scuba Diving or snorkelling in early September 2013 by gently hand collecting macroalgae, with which the annelids are associated inside fabric bags, from either two ‘control’ (C) sites (low *p*CO_2_/high pH, hereafter low *p*CO_2_), and one ‘acidified’ (A) site (high *p*CO_2_/low pH, hereafter high *p*CO_2_) around the island of Ischia (Italy) (Figure S1). Control sites were located at 1–2 m deep (i) at the Scogli di Sant’Anna, which is approx. 600 m south of the Castello Aragonese southern vents (40°43’33.33"N, 13°57’36.38"E) (SA in Figure S1C), and (ii) at the Punta San Pietro approx. 4 km north from the northern venting site (40°44′48″N, 13°56′39″E) (C1-C3 in Figure S1D) where pH values are representative of low *p*CO_2_ conditions (mean pH 8.13 ± 0.01)^62,76^. The ‘acidified’ site of collection was located 1–2 m deep on a rocky reef in an area with high *p*CO_2_/low pH conditions caused by CO_2_ venting at the south side of the Castello Aragonese (40°43′53″N, 13°57′47″E) (mean pH 7.4 to 7.9, A1-A2 in Figure S1C)^18^. Water temperature at the time of collection and exposure varied between 25.5 and 26.8 °C, based on a temperature logger (HOBO sensor, Pendant UA002, Onset, Bourne, Massachusetts, USA) at the south vents (mean T, 1 st −9th September 2013: 26.0 ± 0.3 °C, Gambi M.C. and Lorenti L. unpublished data). Results for the carbonate system (Table S1) confirmed that pH and *p*CO_2_ differed significantly outside (C) and inside (A) the CO_2_ vent areas, consistent with previous studies^21,23,24,27,31,32,76^. All specimens were transferred to the Ischia Marine Centre of the Stazione Zoologica Anton Dohrn (Naples) (approx. 4 km from the vents) within 30 min of collection using cool boxes filled with sea water of the appropriate pH from the collection site (approx. vol. = 10 L). The ‘cool box’ volume ensured that changes in sea water temperature, salinity, O_2_ and pH were minimised during transport (confirmed by remeasurement of these parameters in the cool box upon arrival). Once in the laboratory, specimens were sorted from macroalgae, and maintained for two days prior to the experiment in glass bowls (approx. 20 individuals *per* bowl), each containing 300 mL of natural seawater at the original *p*CO_2_/pH which was changed daily to avoid the accumulation of debris and metabolic byproducts. All glass bowls were kept in a temperature control room to maintain stable conditions (T = 19 °C, 12 L:12 D cycle). Each bowl was supplied with a few pieces of macroalgae from the collection site for specimens to attach to and feed upon. There was no mortality of worms in the laboratory for the two- day holding period and they maintained normal feeding and activity levels.

### Experimental design, study area and experimental procedure

In order to characterise the cellular pathways or remodelling underpinning the relative sensitivity to high *p*CO_2_ of the sibling species *P. cf.*. *massiliensis* and *P. cf.*. *dumerilii*, an in situ transplant experiment utilising the CO_2_ vents of Ischia was conducted. Annelids collected from C sites were transplanted to either Punta San Pietro (CC transplant) approx. 4 km from the venting site (40°44′48″N, 13°56′39″E) where pH values are representative of low *p*CO_2_ conditions (mean pH 8.13 ± 0.01^76^, (CC transplant) (Figure S1D) or to the vent A site (CA transplant) (Figure S1C). Specimens were also collected from the A sites and transplanted to the C site of Punta San Pietro (AC transplant) (Figure S1D) or to the vent A site (AA transplant) (Figure S1C).

In each deployment site, three stations were identified, approx. 50 m from each other and designated as C1, C2 and C3 for control (Figure S1D), and A1, A2 and A3 (Figure S1C) for the high *p*CO_2_ vented area^16^ in order to allow for spatial replication. Each station consisted of a weighted line with a buoy (approx. 2.5 m depth) to which the experimental containers or ‘transplantation chambers’ (TCs) could be attached. These were constructed from white PVC tubes (diameter = 4 cm, length = 11 cm) with a nytal plankton net (mesh = 100 µm) fixed to both ends. This net size allowed for the continual flow through of seawater, but at the same time prevented individuals from escaping, being washed away or being predated upon. On the day of deployment, specimens (n = 15 *per* TC) were transferred to the TCs underwater to avoid any contact with air and air conditions. TCs were then transferred (always avoiding air exposure) to tanks (approx. vol. 10 L each, three TCs *per* tank) and transported to the experimental site: directly from land for the control site and *via* boat in the case of the vents site. TCs were then immediately deployed by SCUBA to each station (n = 1–2 TCs per station, Table S2) in both the control and vents areas where they remained for 5 d under natural conditions, using a previously published experimental design^16,24^. Seawater temperature, salinity and pH were measured at each station at the same time each day during the 5 d experimental period, as diel flux was minimal in the abiotic parameters measured^27,66^. Seawater samples were also taken for total alkalinity analyses to be used to reconstruct the experimental sites’ full carbonate system (see Supplementary Materials). After 5 d exposure, TCs were recovered by SCUBA, placed underwater in 10 L tanks containing fresh seawater of the appropriate pH collected from the respective experimental areas in order to avoid any exposure to air. To minimise environmental shocks, specimens were transported to the laboratory within 30 min. Upon arrival in the laboratory, annelids were rapidly and carefully removed from the TCs, immediately Weighed and snap frozen in liquid nitrogen inside 1.5 mL screw cap microcentrifuge tubes and maintained in −80°C freezer, until being shipped to the Marine Biology and Ecology Research Centre (MBERC) at the University of Plymouth (Plymouth, UK) on dry ice for analyses.

At MBERC individuals were randomly assigned to two groups for either (i) proteomic analyses (*n* = 3), or (ii) metabolomics (including lipidomics) (*n* = 7–8) (see Table S12 for details). Prior to these analyses, a snippet of tissue (the last few segments of the posterior body) was taken from each individual under liquid nitrogen for genetic barcoding (COI). A third group (*n* = 7–8) was assigned for genetic barcoding only. Whilst it is recognised that *P. cf.*. *massiliensis* predominately colonises high *p*CO_2_ vent areas, and that *P. cf.*. *dumerilii* predominantly inhabits low *p*CO_2_ areas^66^ all individuals were barcoded to confirm their genetic identities as the two species are morphologically identical^95^. Following this, designated specimens were shipped on dry ice to the NBAF metabolomics facility at University of Birmingham (Birmingham, UK) for metabolomics, or to the Swire Institute of Marine Sciences at the University of Hong Kong (Hong Kong, China) for proteomic analyses. For full details of omics and barcoding data gathering and statistical analysis workflows see Supplementary Materials.

## Supplementary Information

Below is the link to the electronic supplementary material.

Supplementary Material 1

Supplementary Material 2

Supplementary Material 3

## Data Availability

The data that support the findings of this study are openly available in PANGAEA at doi: 10.1594/PANGAEA.953826, doi: 10.1594/PANGAEA.953832 and doi: 10.1594/PANGAEA.953906, MassIVE https://massive.ucsd.edu, reference number MSV000091611 doi:10.25345/C57W67G3J, and GenBank https://www.ncbi.nlm.nih.gov/genbank, reference numbers ON964737-ON964822.
