# Supplementary material for: Sibling species differently distributed around a CO2 vent show transplantation proteomic remodelling, while displaying metabolomic signatures associated with their origin
Source: Sci Rep. 2025 Sep 29;15:33604. doi: 10.1038/s41598-025-18913-y (PMC12479771; doi:10.1038/s41598-025-18913-y)
Supplement: Supplementary file 1 — Supplementary Material 1 [file 41598_2025_18913_MOESM1_ESM.docx]

**Supplementary materials**

**Additional details for Materials and Methods**

**Environmental monitoring and profiles**

Seawater temperature, salinity and pH were measured at each station daily during the 5 d experimental period using a digital thermometer with thermocouple (HH806AU, Omega Ltd., Manchester, UK), a refractometer (TA 197 LFMulti350, WTW, Weilheim, Germany) and a pH microelectrode (Seven Easy pH InLab, Mettler-Toledo Ltd., Beaumont Leys, UK), maintained at ambient seawater temperature, coupled to a pH meter (Sevengo, Mettler-Toledo Ltd.), calibrated using pH standards (pH 4.01, 7.00, 9.21 at 25 °C, Mettler-Toledo Ltd.) and also maintained at ambient seawater temperature. To determine sea water total alkalinity (TA), samples of sea water (vol. = 100 mL) were also collected at each station daily using Kevlar bottles with a secure tight lid. These were transported inside a cool box to the laboratory and poisoned upon arrival with HgCl_2_ within approximately 1 h from collection. Samples were subsequently shipped to the laboratory of the Marine Biology and Ecology Research Centre (MBERC) (Plymouth, UK) where TA was determined using an alkalinity titrator (AS-ALK2, Apollo SciTech, Bogart, USA).

Seawater dissolved inorganic carbon (DIC), partial pressure of CO_2_ (*p*CO_2_), calcite and aragonite saturation (*Ω*_calc_ and *Ω*_ara_), bicarbonate and carbonate ion concentration ([HCO_3_^–^] and [CO_3_^2–^], respectively) were calculated from pH and TA measurements using the software program CO2SYS (Pierrot, 2006) with dissociation constants from (Mehrbach, 1973) refit by (Dickson, 1987) and [KSO_4_] using (Dickson, 1990).

**Metabolomics including lipidomics**

The mass spectrometry based metabolomics workflow used here has been previously summarised (Kirwan et al. 2014). In brief, 128 μL methanol (HPLC grade) and 51.2 μL water (HPLC grade) per mg wet weight were added to each sample prior to homogenisation using a Precellys-24 ceramic bead-based homogenizer (Stretton Scientific Ltd., UK). Thereafter, 64 μL water (HPLC grade) and 128 μL chloroform (pesticide analysis grade) per mg were added to each sample, which was vortexed and centrifuged (1,800 rcf, 10 min, 4 °C), yielding an upper (polar) and lower (nonpolar) fraction for each sample. Each polar fraction was collected into a 1.5 mL Eppendorf tube, and the nonpolar fraction into a 1.8 mL glass vial. Extraction blanks were also prepared using identical methods except that no biological material was added to the solvents. Two quality control (QC) samples were prepared by pooling small aliquots from the polar and nonpolar extracts, respectively. All polar samples were dried in a centrifugal concentrator (Thermo Savant, Holbrook, NY), the nonpolar samples under a stream of nitrogen gas. All samples were stored at −80 °C prior to analysis.

Direct infusion mass spectrometry (DIMS) data acquisition in the negative ion mode was performed on a Fourier transform ion cyclotron resonance (FT-ICR) mass spectrometer (LTQ FT Ultra, Thermo Fisher Scientific, Bremen, Germany) with a chip-based Triversa nanoelectrospray source (Advion Biosciences, Ithaca, NY). The polar extracts were each taken up in 80-160 μL (double the extract volume) of 20 mM ammonium acetate in methanol/water (4:1 v/v). Samples were centrifuged (22,000 rcf, 10 min, 4 °C) to remove any particulate matter. They were analysed in a controlled-randomised sequence different from the metabolite extraction sequence, with three technical replicates measured for each sample. QC samples were analysed at the beginning, the end, and equidistantly throughout the sequence. Data were acquired at nominal 1E5 resolution from m/z 70–590, in seven wide-SIM (selected ion monitoring) windows of 100 Da. Nonpolar (lipid) samples were taken up in 80-160 μL (double the extract volume) of 5 mM ammonium acetate in methanol/water (4:1 v/v). Data were acquired in increasing SIM windows between 120 and 200 Da width, from m/z 120 to 1600. The nonpolar QC sample was used in the same way as for the polar analyses. The nonpolar samples were also analysed in a controlled-randomised order and each was measured in three technical replicates. Raw mass spectral data were processed using the SIM-stitching algorithm, using an in-house Matlab script. The data matrices were normalized using the PQN algorithm. Missing values were imputed using the KNN algorithm. The resulting data matrix was analysed using univariate statistics, described below. The same matrix was transformed using the generalised logarithm to stabilise the technical variance across the measured peaks prior to analysis using multivariate statistics.

Signals were putatively annotated with empirical formulae calculated by the MIPack software (Weber et al. 2010), searching the KEGG (Kanehisa et al. 2012, 2025) and LipidMaps (Fahy et al. 2007) databases, and confirmed by performing calculations based on the original spectra in Xcalibur 2.0.7 (Thermo Fisher Scientific).

**Proteomic profiling**

The whole animal tissue from the respective samples were sonicated in HEPES buffer (8 M urea, protease inhibitor cocktail, 40 mM HEPES (pH 8.0)) using Branson sonifier for 15 sec pulse at setting 3 for six times with 2 min ice intervals. The supernatant obtained after centrifugation at 14,000 rpm for 15 min at 15 °C were quantified using RC DC assay kit (Biorad, Hercules, CA, USA). The protein samples were cleaned with 2D clean up kit (Biorad, Hercules, CA, USA) prior to trypsin digestion. Then 20µg of protein sample were reduced with 10mM Dithiothreitol DTT for 45 min at 56 °C followed by alkylation using 55 mM Iodoacetamide IAA for 30 min in dark at room temperature. The samples were digested overnight (16 hours) at 37 °C with sequencing grade Trypsin (Promega, Madison, WI, USA) with an enzyme-protein ratio of 1:25. Trypsin digestion was stopped with 1 µl of formic acid diluted to 1 mL of milliQ water and stored at -20 °C until desalting. The tryptic digests were desalted using Sep-Pak C18 cartridges (Waters, Milford, USA). The desalted peptides were vacuum dried (SpeedVac, Thermo Electron, Waltham, USA) and submitted to further mass spectrometric analysis.

***LC-MS/ MS Triple TOF analysis***

Each trypsin digested sample was dissolved in water and subjected to a reversed phase nano-LC-MS/MS consisting of a nano pump equipped with a 10-well-plate auto-sampler (Agilent Technologies, Wilmington, DE, USA) coupling with Triple TOF 5600 system (AB SCIEX, Concord, ON, Canada) fitted with a Nanospray III source (AB SCIEX, Concord, ON, Canada). Peptide elution was applied with a 5-35% ACN gradient in 0.5% formic acid during 180 min gradient after loading on a 5cm reverse-phase C18 trap column. The parameters of MS used were as follows: ion spray voltage, 2.8 kV; curtain gas, 20 psi; nebulizer gas, 6 psi; interface heater temperature, 125℃. For IDA, full scans were acquired within 250 ms over the range m/z 400-1,250, followed by MS/MS scans of the 20 most abundant peaks that exceeded 125 counts per second and carried a charge between +2 to +5 in the range m/z 100—1,500. The dynamic exclusion time of the acquired ions was set at 20 s.

**Sequence generation**

Total genomic DNA was isolated using a modified CTAB protocol (Sokolov 2000). Following DNA extraction, a fragment (~600 base pairs) of the COI gene was amplified using two universal primers (that is, LCOI490, 5’-GTCAACAAATCATAAAGATATTGG-3’ and HCO2198, 5’-TAAACTTCAGGGTGACCAAAAAATCA-3’) (Folmer et al. 1994) using a standard PCR protocol. Sequencing was carried out using the same primers as the initial PCR reactions by Macrogen Inc. (Amsterdam, The Netherlands). Both forward and reverse sequences were generated.

**Statistical analysis**

**Processed metabolomics data**

For both polar and lipid extracts, first processed metabolomics data were analysed using Perseus (Tyanova et al., 2016) and MetaboAnalyst 4.0 (Chong et al., 2019). Principal components analysis (PCA) was used to unravel the data structure and any group separations. Nonparametric PERMutational Multivariate ANalysis Of VAriance tests (PERMANOVAs) were run to determine any differences between treatment groups (CC, CA, AC, AA), regime of origin (C vs. A), and transplant type (SE vs. DE) (Table S4). Due to the fact that not all species were present in all treatment groups (e.g. regime of origin and transplant type) it was not possible to run a PERMANOVA with three factors (e.g. *P.* c.f. *dumerilii*, *P.*c.f. *massiliensis*, and an unknown *Platynereis* sp.). A sample cluster analysis was performed using Euclidean distance and Ward’s clustering algorithm. At this point three outlying samples were identified and removed from further analyses in the polar extracts dataset. Volcano plots were used in Perseus to identify differentially abundant metabolites between (i) individuals transplanted to the same environment (SE, which includes CC and AA) vs. those transplanted to a different environment (DE, which includes CA and AC) and (ii) individuals from the control regime of origin (CC and CA) vs. individuals from the acidified regime of origin (AA and AC). Volcano plots were based on t-tests with 250 randomizations, FDR 0.05 and s 0.1. Next, a heatmap and hierarchical cluster analysis was carried out after Z-scoring the data matrix. Spearman correlation was used as distance measure and complete linkage as clustering method.

**Polar extracts**

To identify enriched compound types and relevant biological pathways within the significantly different m/z peaks, we also applied the mummichog and GSEA algorithms to the data, using the module ‘MS peaks to pathways’ in MetaboAnalyst 4.0 to predict network activity from untargeted metabolome data (see Li et al., 2013). Data was input as a table with m/z peaks, p-values and fold-changes. The following parameters were used in the analysis: molecular weight tolerance 5 ppm, negative mode, p-value <0.01 for peak significance in mummichog, database ‘non-lipids – sub chemical class’, which contains 778 main non-lipid chemical class metabolite sets from RefMet. The same analysis was then run using the KEGG database for the model species Caenorhabditis elegans Maupas 1900, for pathway identification. Significantly enriched compound classes and pathways were based on p < 0.05.

**Lipid extracts**

To identify relevant biological pathways within the significantly different m/z peaks, the ions with a putative annotation were categorized into a main lipid class, and the biological function of each lipid class was retrieved from the Encyclopedia of Lipidomics (Wenk, 2019) and HMDB Metabocards. As lipid annotation is still a major bottleneck in untargeted lipidomics, we also applied the mummichog and GSEA algorithms to the data, using the module ‘MS peaks to pathways’ in Metaboanalyst 4.0 to predict network activity from untargeted lipidome data, bypassing the need to identify lipids (see Li et al., 2013). Data was input as a table with m/z peaks, p-values and fold-changes. The following parameters were used in the analysis: molecular weight tolerance 5 ppm, negative mode, p-value <0.01 for peak significance in mummichog, database ‘lipids – main chemical class’, which contains 77 main lipid chemical class metabolite sets from RefMet. Significantly enriched lipid classes were based on p<0.05.

**Proteomics data**

The acquired MS/MS raw data were processed using the Label-free quantification (LFQ) analysis by public domain software suites such as MaxQuant (Cox and Mann, 2008). Mass spectrometric data were searched against the sequence database consisting of the predicted *Platynereis* proteome (51,767 entries) (Conzelmann et al., 2013). Proteomic data were analysed in the free software Perseus (Tyanova et al., 2016) and MetaboAnalyst 4.0 (Chong et al., 2019). Proteomic data were pre-processed in Perseus based on three steps (i) filtering out rows with < 60 % valid values, (ii) log2 transformation of the data, (iii) data normalization by Z-scoring the matrix. Following, missing data imputation was done by replacing missing values from a normal distribution (width 0.3, down shift 1.8, separately for each sample).

A Principal Components Analysis (PCA) was then carried out in MetaboAnalyst to unravel data structure and group separation. PERMANOVAs were run to determine any differences between treatment groups (CC, CA, AC, AA), regime of origin (C vs. A), and transplant type (SE vs. DE) (Table S4). A sample cluster analysis was performed using Euclidean distance and Ward’s clustering algorithm. Following, a heatmap and hierarchical cluster analysis was carried out in Perseus using Spearman correlation as distance and complete linkage as clustering method for proteins. Profile plots of the protein relative abundances across samples were also plotted for each cluster. A volcano plot was used to identify differentially abundant proteins (DAPs) between (i) transplant type, namely individuals transplanted to the same environment (SE, which includes CC and AA) vs. transplanted to a different environment (DE, which includes CA and AC) and (ii) regime of origin (control vs. acidified) based on t-tests with 250 randomizations, FDR 0.05 and s 0.1. Then, the DAPs were used as input list to carry out a protein network analysis in Cytoscape v3.8.0 (plugins ClueGO v2.5.7 and CluePedia v1.5.7). As information on *Platynereis* spp. is not available on the software, the analysis was carried out using the model Caenorhabditis elegans with the following parameters - ontologies: GO_ImmuneSystemProcess-GOA_17.11.2016_10h53, KEGG_17.11.2016, GO_BiologicalProcess-GOA_17.11.2016_10h53, enrichment/depletion: two-sided hypergeometric with Bonferroni step down correction and p-value of 0.05, GO level 3 to 8, minimum number of genes 1, minimum percentage of genes 4 %, Kappa score 0.4). Protein function was further validated via search in UniProt and QuickGO.

**Phylogenetic analysis**

Sequences were confirmed as *Platynereis* spp. using the Gen-Bank BLASTn search. All sequences were then aligned using ClustalW and edited using BIOEDIT 7.0.5.3 (Hall 1999). All alignments and base substitutions were confirmed visually. Phylogenetic reconstruction was carried out using maximum likelihood (ML) in MEGA X (Kumar et al. 2018). Prior to ML analysis, MODELTEST version 3.07 (Posada and Crandall 1998) was used to select the most appropriate model of sequence evolution using the Akaike information criterion (AIC). All other settings were left as defaults. Support was measured with 1,000 bootstrap replicates. Only clades with significant support values (defined here as those with >70 % bootstrap support) are shown. To further investigate genetic differentiation among individuals sampled from inside or outside the high pCO2 vent area, sequences were collapsed into haplotypes using DnaSP version 6.12.03 (Rozas et al. 2017). Sequence divergences (uncorrected p-distance) between haplotypes, and the vent inhabiting *P. massiliensis* ecotype characterised and sequenced by Lucey et al. (2015) and designated as a template sequence (GenBank accession number KP127953) were calculated using Arlequin version 3.5.2.2. (Excoffier et al. 2005). Evolutionary relationships among haplotypes (gene genealogies) were analysed through the construction of a haplotype network. Beforehand, additional *P.* c.f. *massiliensis* and *P.* c.f. *dumerilii* COI sequences were downloaded from GenBank to use as reference sequences and added to the dataset in a preliminary analysis to assess sequence species identity (Table S3). The R package pegas v1.1 (Paradis, 2010) run in R (R Statistical Software v4.1.2; R Core Team 2021) was used to produce a haplotype network with maximum parsimony then applied to infer the most parsimonious branch connections between the haplotypes detected among all the sites sampled (Templeton et al. 1992).

**Additional details for Results**

**Table S1.** Summary of the mean (± SEM) and calculated physico-chemical parameters of sea water used when exposing worms to: (i) current *p*CO_2_/pH conditions (‘control’ stations C1, C2 and C3) and (ii) elevated *p*CO_2_/low pH conditions (‘acidified’ stations A1, A2, A3). Salinity, temperature, pH_NBS_ (Mettler-Toledo pH meter, Beaumont Leys, UK), total alkalinity (TA) (AS-ALK2, Apollo SciTech, Bogart, USA), dissolved inorganic carbon (DIC), carbon dioxide partial pressure (*p*CO_2_), bicarbonate and carbonate ion concentration ([HCO_3_^–^] and [CO_3_^2–^), calcite and aragonite saturation state (Ωcal and Ωara) are provided. The significant difference in temperature between the two sites was negligible and within the range of natural fluctuation experienced at these sites*.*

| **Parameter** | **Control** | | | | **Acidified** | | | |
| --- | --- | --- | --- | --- | --- | --- | --- | --- |
|  | **C1** | **C2** | **C3** | **Overall** | **A1** | **A2** | **A3** | **overall** |
| **Salinity** | 36.93 ± 0.11 | 37.00 ± 0.10 | 37.02 ± 0.09 | 36.98 ± 0.06 | 37.00 ± 0.08 | 37.08 ± 0.09 | 37.05 ± 0.12 | 37.04 ± 0.06 |
| **Temperature (°C)** | 21.29 ± 0.30^A^ | 21.36 ± 0.32^A^ | 21.37 ± 0.31^A^ | 21.34 ± 0.18^a^ | 20.17 ± 0.18^B^ | 20.12 ± 0.18^B^ | 20.41 ± 0.16^B^ | 20.23 ± 0.10^b^ |
| **pH** | 8.12 ± 0.01^A^ | 8.13 ± 0.01^A^ | 8.15 ± 0.01^A^ | 8.13 ± 0.01^a^ | 7.49 ± 0.06^B^ | 7.16 ± 0.08^C^ | 7.21 ± 0.06^C^ | 7.29 ± 0.04^b^ |
| **TA (µequiv kg^-1^)** | 2612.93 ± 4.68 | 2589.79 ± 6.92 | 2600.38 ± 4.05 | 2601.33 ± 3.21 | 2620.00 ± 4.19 | 2597.40 ± 11.21 | 2606.72 ± 14.29 | 2608.15 ± 6.22 |
| **DIC (µmol kgˉ¹)^*^** | 2333.39 ± 9.19^A^ | 2305.76 ± 13.71^A^ | 2305.98 ± 9.62^A^ | 2315.40 ± 6.39^a^ | 2640.00 ± 31.65^B^ | 2802.89 ± 47.34^C^ | 2759.76 ± 41.80^C^ | 2733.52 ± 24.25^b^ |
| ***p*CO2 (µatm) ^*^** | 518.05 ± 13.50^A^ | 505.30 ± 19.00^A^ | 482.89 ± 14.19^A^ | 502.24 ± 9.05^a^ | 3682.36 ± 702.65^B^ | 8608.77 ± 1150.84^C^ | 6216.07 ± 798.54^D^ | 6144.17 ± 552.63^b^ |
| **[HCO_3_^-^] (µmol kgˉ¹) ^*^** | 2109.27 ± 12.82^A^ | 2079.87 ± 18.26^A^ | 2073.80 ± 13.79^A^ | 2088.02 ± 8.73^a^ | 2455.28 ± 20.62^B^ | 2481.77 ± 27.81^B^ | 2509.83 ± 23.58^B^ | 2482.30 ± 13.96^b^ |
| **[CO_3_^2-^] (µmol kgˉ¹) ^*^** | 208.07 ± 4.54^A^ | 210.26 ± 5.61^A^ | 217.29 ± 4.98^A^ | 211.84 ± 2.90^a^ | 68.08 ± 7.79^B^ | 47.76 ± 11.14^C^ | 40.02 ± 6.50^C^ | 52.00 ± 5.08^b^ |
| **Ω^b^cal** | 4.91 ± 0.11^A^ | 4.96 ± 0.13^A^ | 5.12 ± 0.12^A^ | 5.00 ± 0.07^a^ | 1.60 ± 0.18^B^ | 1.12 ± 0.26^C^ | 0.94 ± 0.15^C^ | 1.22 ± 0.12^b^ |
| **Ω^b^ara** | 3.21 ± 0.07^A^ | 3.24 ± 0.09^A^ | 3.35 ± 0.08^A^ | 3.27 ± 0.05^a^ | 1.05 ± 0.12^B^ | 0.73 ± 0.17^C^ | 0.61 ± 0.10^C^ | 0.80 ± 0.08^b^ |

Different capital letters (A,B,C,D) indicate significant differences of the mean values of a parameter from different stations.

Different lowercase letters (a,b) indicate significant differences of the mean values of a parameter from different treatments.

^*^Parameters that were calculated using the CO2SYS program (Pierrot et al., 2006), using the dissociation constants of Mehrbach et al. (1973) as refitted by Dickson & Millero (1987) and [KSO4] using Dickson (1990).

**Table S2.** Summary of the survival rates for *Platynereis* spp. after exposure to control or acidified conditions.

| **Treatment** | **Station** | **Transplantation chamber** | **% survival** |
| --- | --- | --- | --- |
| C-C | C1 | A | 46.67 |
|  |  | B | 13.33 |
|  | C2 | A | 53.33 |
|  | C3 | A | 26.67 |
| C-A | A1 | A | 33.33 |
|  |  | B | 33.33 |
|  | A2 | A | 33.33 |
|  | A3 | A | 73.33 |
| A-C | C1 | A | 80.00 |
|  |  | B | 80.00 |
|  | C2 | A | 60.00 |
|  | C3 | A | 20.00 |
| A-A | A1 | A | 0 |
|  |  | B | 60.00 |
|  | A2 | A | 66.67 |
|  | A3 | A | 40.00 |

**Table S3.** List of haplotypes and individual samples of *Platynereis* sp. with Genbank accession numbers and source of the sequences. Species specified where confirmed based on reproductive traits.

| **Haplotype** | **Sample ID/ species** | **Collection location** | **GenBank Accession** | **Reference** |
| --- | --- | --- | --- | --- |
| Platy 1 | CA61  CA62  CA66  CA67  CA68  CC171  CC176  CC177  CC178  CC185  CC186  CC187  CC190  AA445  AA447  AA448  AA458  AA460  AA461  AC563  AC572  AC574  AC584  AC587  *P. dumerilii*  *Platynereis* sp. | control (Ischia, Italy)  control (Ischia, Italy)  control (Ischia, Italy)  control (Ischia, Italy)  control (Ischia, Italy)  control (Ischia, Italy)  control (Ischia, Italy)  control (Ischia, Italy)  control (Ischia, Italy)  control (Ischia, Italy)  control (Ischia, Italy)  control (Ischia, Italy)  control (Ischia, Italy)  acidified (Ischia, Italy)  acidified (Ischia, Italy)  acidified (Ischia, Italy)  acidified (Ischia, Italy)  acidified (Ischia, Italy)  acidified (Ischia, Italy)  acidified (Ischia, Italy)  acidified (Ischia, Italy)  acidified (Ischia, Italy)  acidified (Ischia, Italy)  acidified (Ischia, Italy)  control (Ischia, Italy) | ON964737  ON964738  ON964740  ON964741  ON964742  ON964754  ON964758  ON964759  ON964760  ON964766  ON964767  ON964768  ON964770  ON964773  ON964775  ON964776  ON964783  ON964785  ON964786  ON964797  ON964806  ON964809  ON964817  ON964820  KP127954  ON716019 | this study  this study  this study  this study  this study  this study  this study  this study  this study  this study  this study  this study  this study  this study  this study  this study  this study  this study  this study  this study  this study  this study  this study  this study  Lucey et al. (2015)  unpublished |
|  | *Platynereis* sp.  *Platynereis* sp.  *Platynereis* sp.  *Platynereis* sp. | Arcachon (Atlantic, France)  Arcachon (Atlantic, France)  Arcachon (Atlantic, France)  Arcachon (Atlantic, France) | KT124668  KT124669  KT124670  KT124671 | Wäge et al. (2017)  Wäge et al. (2017)  Wäge et al. (2017)  Wäge et al. (2017) |
| Platy 2 | *Platynereis* sp.  *Platynereis* sp.  *Platynereis* sp.  *Platynereis* sp.  *Platynereis* sp.  *Platynereis* sp.  *Platynereis* sp.  *Platynereis* sp.  *Platynereis* sp.  *Platynereis* sp.  *Platynereis* sp.  *Platynereis* sp.  *Platynereis* sp.  *Platynereis* sp.  *Platynereis* sp.  *Platynereis* sp.  *Platynereis* sp.  *Platynereis* sp.  *Platynereis* sp.  *Platynereis* sp.  *Platynereis* sp.  *Platynereis* sp.  *Platynereis* sp.  *Platynereis* sp.  *Platynereis* sp.  *Platynereis* sp.  *Platynereis* sp.  *Platynereis* sp.  *Platynereis* sp.  *Platynereis* sp.  *Platynereis* sp.  *Platynereis* sp.  *Platynereis* sp.  *Platynereis* sp.  *Platynereis* sp.  *Platynereis* sp.  *Platynereis* sp.  *Platynereis* sp.  *Platynereis* sp.  *Platynereis* sp.  *Platynereis* sp.  *Platynereis* sp.  *Platynereis* sp.  *Platynereis* sp.  *Platynereis* sp.  *Platynereis* sp.  *Platynereis* sp.  *Platynereis* sp.  *Platynereis* sp.  *Platynereis* sp.  *Platynereis* sp.  *Platynereis* sp.  *Platynereis* sp.  *Platynereis* sp.  *Platynereis* sp.  *Platynereis* sp.  *Platynereis* sp.  *Platynereis* sp.  *Platynereis* sp.  *Platynereis* sp.  *Platynereis* sp.  *Platynereis* sp.  *Platynereis* sp.  *Platynereis* sp.  *Platynereis* sp.  *Platynereis* sp.  *Platynereis* sp.  *Platynereis* sp.  *Platynereis* sp.  *Platynereis* sp.  *Platynereis* sp.  *Platynereis* sp.  *Platynereis* sp.  *Platynereis* sp.  *Platynereis* sp.  CA63  CA74  CA75  CA76  CC172  CC180  CC182  CC183  AA459  AA464  AC566  AC583  *Platynereis* sp.  *Platynereis* sp.  *Platynereis* sp.  *Platynereis* sp.  *Platynereis* sp.  *Platynereis* sp.  *Platynereis* sp.  *Platynereis* sp.  *Platynereis* sp.  *Platynereis* sp.  *Platynereis* sp.  *Platynereis* sp. | Arcachon (Atlantic, France)  Arcachon (Atlantic, France)  Blanes (Catalunia, Spain)  Blanes (Catalunia, Spain)  Blanes (Catalunia, Spain)  Blanes (Catalunia, Spain)  Blanes (Catalunia, Spain)  Ischia, Italy  Ischia, Italy  Ischia, Italy  Ischia, Italy  Ischia, Italy  Ischia, Italy  Ischia, Italy  Ischia, Italy  Palinuro (Tyrrhenian Sea)  Palinuro (Tyrrhenian Sea)  Palinuro (Tyrrhenian Sea)  Palinuro (Tyrrhenian Sea)  Calvi (Corsica, France)  Palinuro (Tyrrhenian Sea)  Palinuro (Tyrrhenian Sea)  Palinuro (Tyrrhenian Sea)  control (Ischia, Italy)  control (Ischia, Italy)  control (Ischia, Italy)  control (Ischia, Italy)  control (Ischia, Italy)  control (Ischia, Italy)  control (Ischia, Italy)  control (Ischia, Italy)  control (Ischia, Italy)  control (Ischia, Italy)  control (Ischia, Italy)  control (Ischia, Italy)  acidified (Ischia, Italy)  Nisida, Italy  Nisida, Italy  Nisida, Italy  Nisida, Italy  Nisida, Italy  Nisida, Italy  Nisida, Italy  Nisida, Italy  Nisida, Italy  Nisida, Italy  control (Ischia, Italy)  control (Ischia, Italy)  control (Ischia, Italy)  control (Ischia, Italy)  control (Ischia, Italy)  control (Ischia, Italy)  control (Ischia, Italy)  control (Ischia, Italy)  control (Ischia, Italy)  Forio (Naples, Italy)  Forio (Naples, Italy)  Forio (Naples, Italy)  Forio (Naples, Italy)  Forio (Naples, Italy)  Forio (Naples, Italy)  Bristol Channel, UK  Bristol Channel, UK  Bristol Channel, UK  Bristol Channel, UK  Bristol Channel, UK  Bristol Channel, UK  Bristol Channel, UK  Bristol Channel, UK  Bristol Channel, UK  Bristol Channel, UK  Bristol Channel, UK  Bristol Channel, UK  Bristol Channel, UK  Bristol Channel, UK  control (Ischia, Italy)  control (Ischia, Italy)  control (Ischia, Italy)  control (Ischia, Italy)  control (Ischia, Italy)  control (Ischia, Italy)  control (Ischia, Italy)  control (Ischia, Italy)  acidified (Ischia, Italy)  acidified (Ischia, Italy)  acidified (Ischia, Italy)  acidified (Ischia, Italy)  Ischia, Italy  Ischia, Italy  Ischia, Italy  Ischia, Italy  Ischia, Italy  Ischia, Italy  Ischia, Italy  Ischia, Italy  Ischia, Italy  Ischia, Italy  Ischia, Italy  acidified (Ischia, Italy) | KT124672  KT124673  KT124675  KT124676  KT124677  KT124678  KT124679  KT124685  KT124686  KT124687  KT124688  KT124689  KT124690  KT124691  KT124692  KT124696  KT124698  KT124700  KT124701  KT124703  KT124706  KT124707  KT124708  KC591812  KC591813  KC591814  KC591815  KC591816  KC591817  KC591818  KC591820  KC591821  KC591823  KC591824  KC591825  KC591837  KC591839  KC591840  KC591842  KC591843  KC591845  KC591846  KC591847  KC591850  KC591851  KC591852  KC591856  KC591857  KC591858  KC591859  KC591862  KC591863  KC591864  KC591865  KC591867  KC591877  KC591878  KC591879  KC591881  KC591882  KC591883  KC591884  KC591885  KC591886  KC591887  KC591888  KC591889  KC591890  KC591891  KC591892  KC591893  KC591894  KC591896  KC591897  KC591898  ON964739  ON964746  ON964747  ON964748  ON964755  ON964762  ON964763  ON964764  ON964784  ON964788  ON964800  ON964816  OQ673199  OQ673201  OQ673202  OQ673203  OQ673204  OQ673205  OQ673207  OQ673208  OQ673209  OQ673210  OQ673212  KC591835 | Wäge et al. (2017)  Wäge et al. (2017)  Wäge et al. (2017)  Wäge et al. (2017)  Wäge et al. (2017)  Wäge et al. (2017)  Wäge et al. (2017)  Wäge et al. (2017)  Wäge et al. (2017)  Wäge et al. (2017)  Wäge et al. (2017)  Wäge et al. (2017)  Wäge et al. (2017)  Wäge et al. (2017)  Wäge et al. (2017)  Wäge et al. (2017)  Wäge et al. (2017)  Wäge et al. (2017)  Wäge et al. (2017)  Wäge et al. (2017)  Wäge et al. (2017)  Wäge et al. (2017)  Wäge et al. (2017)  Calosi et al. (2013)  Calosi et al. (2013)  Calosi et al. (2013)  Calosi et al. (2013)  Calosi et al. (2013)  Calosi et al. (2013)  Calosi et al. (2013)  Calosi et al. (2013)  Calosi et al. (2013)  Calosi et al. (2013)  Calosi et al. (2013)  Calosi et al. (2013)  Calosi et al. (2013)  Calosi et al. (2013)  Calosi et al. (2013)  Calosi et al. (2013)  Calosi et al. (2013)  Calosi et al. (2013)  Calosi et al. (2013)  Calosi et al. (2013)  Calosi et al. (2013)  Calosi et al. (2013)  Calosi et al. (2013)  Calosi et al. (2013)  Calosi et al. (2013)  Calosi et al. (2013)  Calosi et al. (2013)  Calosi et al. (2013)  Calosi et al. (2013)  Calosi et al. (2013)  Calosi et al. (2013)  Calosi et al. (2013)  Calosi et al. (2013)  Calosi et al. (2013)  Calosi et al. (2013)  Calosi et al. (2013)  Calosi et al. (2013)  Calosi et al. (2013)  Calosi et al. (2013)  Calosi et al. (2013)  Calosi et al. (2013)  Calosi et al. (2013)  Calosi et al. (2013)  Calosi et al. (2013)  Calosi et al. (2013)  Calosi et al. (2013)  Calosi et al. (2013)  Calosi et al. (2013)  Calosi et al. (2013)  Calosi et al. (2013)  Calosi et al. (2013)  Calosi et al. (2013)  this study  this study  this study  this study  this study  this study  this study  this study  this study  this study  this study  this study  unpublished  unpublished  unpublished  unpublished  unpublished  unpublished  unpublished  unpublished  unpublished  unpublished  unpublished  Calosi et al. (2013) |
| Platy 3 | CA71  AA467  AC567  AC568  *Platynereis* sp.  *Platynereis* sp.  *Platynereis* sp.  *Platynereis* sp. | control (Ischia, Italy)  acidified (Ischia, Italy)  acidified (Ischia, Italy)  acidified (Ischia, Italy)  control (Ischia, Italy)  control (Ischia, Italy)  control (Ischia, Italy)  Forio (Naples, Italy) | ON964743  ON964791  ON964801  ON964802  KC591854  KC591855  KC591866  KC591880 | this study  this study  this study  this study  Calosi et al. (2013)  Calosi et al. (2013)  Calosi et al. (2013)  Calosi et al. (2013) |
| Platy 4  Platy 5 | CA72  CA73  CC189  AA466  *Platynereis* sp.  *Platynereis* sp. | control (Ischia, Italy)  control (Ischia, Italy)  control (Ischia, Italy)  acidified (Ischia, Italy)  Ischia, Italy  Ischia, Italy | ON964744  ON964745  ON964769  ON964790  OQ673206  OQ673200 | this study  this study  this study  this study  unpublished  unpublished |
| Platy 6  Platy 7  Platy 8  Platy 9  Platy10  Platy11  Platy12  Platy13  Platy14  Platy15  Platy16  Platy17  Platy18 | CA79  CA80  CA82  CA83  AA443  AA444  AA446  AA451  AA452  AA453  AA454  AA456  AA457  AA463  AA465  AC558  AC559  AC560  AC561  AC564  AC565  AC569  AC570  AC571  AC572A  AC573  AC575  AC576  AC581  AC582  AC585  AC586  AC592  *P. massiliensis*  *Platynereis* sp.  *Platynereis* sp.  *Platynereis* sp.  *Platynereis* sp.  *Platynereis* sp.  *Platynereis* sp.  *Platynereis* sp.  *Platynereis* sp.  *Platynereis* sp.  *Platynereis* sp.  *Platynereis* sp.  *Platynereis* sp.  *Platynereis* sp.  *Platynereis* sp.  *Platynereis* sp.  CC170  CC173  CC174  CC179  CC184  AC562  *Platynereis* sp.  *Platynereis* sp.  *Platynereis* sp.  *Platynereis* sp.  *Platynereis* sp.  *Platynereis* sp.  *Platynereis* sp.  *Platynereis* sp.  *Platynereis* sp.  *Platynereis* sp.  *Platynereis* sp.  AC577  AC580  AC591 | control (Ischia, Italy)  control (Ischia, Italy)  control (Ischia, Italy)  control (Ischia, Italy)  acidified (Ischia, Italy)  acidified (Ischia, Italy)  acidified (Ischia, Italy)  acidified (Ischia, Italy)  acidified (Ischia, Italy)  acidified (Ischia, Italy)  acidified (Ischia, Italy)  acidified (Ischia, Italy)  acidified (Ischia, Italy)  acidified (Ischia, Italy)  acidified (Ischia, Italy)  acidified (Ischia, Italy)  acidified (Ischia, Italy)  acidified (Ischia, Italy)  acidified (Ischia, Italy)  acidified (Ischia, Italy)  acidified (Ischia, Italy)  acidified (Ischia, Italy)  acidified (Ischia, Italy)  acidified (Ischia, Italy)  acidified (Ischia, Italy)  acidified (Ischia, Italy)  acidified (Ischia, Italy)  acidified (Ischia, Italy)  acidified (Ischia, Italy)  acidified (Ischia, Italy)  acidified (Ischia, Italy)  acidified (Ischia, Italy)  acidified (Ischia, Italy)  acidified (Ischia, Italy)  acidified (Ischia, Italy)  acidified (Ischia, Italy)  acidified (Ischia, Italy)  acidified (Ischia, Italy)  control (Ischia, Italy)  acidified (Ischia, Italy)  acidified (Ischia, Italy)  acidified (Ischia, Italy)  acidified (Ischia, Italy)  acidified (Ischia, Italy)  acidified (Ischia, Italy)  acidified (Ischia, Italy)  acidified (Ischia, Italy)  acidified (Ischia, Italy)  acidified (Ischia, Italy)  control (Ischia, Italy)  control (Ischia, Italy)  control (Ischia, Italy)  control (Ischia, Italy)  control (Ischia, Italy)  acidified (Ischia, Italy)  Palinuro (Tyrrhenian Sea)  Palinuro (Tyrrhenian Sea)  Palinuro (Tyrrhenian Sea)  Palinuro (Tyrrhenian Sea)  control (Ischia, Italy)  control (Ischia, Italy)  Nisida, Italy  Nisida, Italy  Nisida, Italy  Nisida, Italy  Nisida, Italy  acidified (Ischia, Italy)  acidified (Ischia, Italy)  acidified (Ischia, Italy) | ON964749  ON964750  ON964751  ON964752  ON964771  ON964772  ON964774  ON964777  ON964778  ON964779  ON964780  ON964781  ON964782  ON964787  ON964789  ON964792  ON964793  ON964794  ON964795  ON964798  ON964799  ON964803  ON964804  ON964805  ON964806  ON964808  ON964810  ON964811  ON964814  ON964815  ON964818  ON964819  ON964822  KP127953  KT124680  KT124681  KT124682  KT124683  KC591822  KC591827  KC591828  KC591829  KC591830  KC591831  KC591832  KC591833  KC591834  KC591836  KC591838  ON964753  ON964756  ON964757  ON964761  ON964765  ON964796  KT124694  KT124695  KT124697  KT124699  KC591819  KC591826  KC591841  KC591844  KC591848  KC591849  KC591853  ON964812  ON964813  ON964821 | this study  this study  this study  this study  this study  this study  this study  this study  this study  this study  this study  this study  this study  this study  this study  this study  this study  this study  this study  this study  this study  this study  this study  this study  this study  this study  this study  this study  this study  this study  this study  this study  this study  Lucey et al. (2015)  Wäge et al. (2017)  Wäge et al. (2017)  Wäge et al. (2017)  Wäge et al. (2017)  Calosi et al. (2013)  Calosi et al. (2013)  Calosi et al. (2013)  Calosi et al. (2013)  Calosi et al. (2013)  Calosi et al. (2013)  Calosi et al. (2013)  Calosi et al. (2013)  Calosi et al. (2013)  Calosi et al. (2013)  Calosi et al. (2013)  this study  this study  this study  this study  this study  this study  Wäge et al. (2017)  Wäge et al. (2017)  Wäge et al. (2017)  Wäge et al. (2017)  Calosi et al. (2013)  Calosi et al. (2013)  Calosi et al. (2013)  Calosi et al. (2013)  Calosi et al. (2013)  Calosi et al. (2013)  Calosi et al. (2013)  this study  this study  this study |
|  |  |  |  |  |

**Table S4.** Results of PERMANOVAs run to determine any differences between treatment groups (CC, CA, AC, AA), site of origin (C vs. A), and transplant type (SE vs. DE) for the polar and lipid metabolomics extracts and proteomics samples.

| **Omics compartment** | **Design** | **df** | **SS** | **MS** | **Pseudo-F** | **P (perm)** | **Unique perms** |
| --- | --- | --- | --- | --- | --- | --- | --- |
| Lipids | Treatments  (CC, CA, AC, AA) | 3 | 2135.1 | 711.7 | 2.138 | **0.0004** | 9873 |
|  | Site of origin  (C *vs.* A) | 1 | 1519.1 | 1519.1 | 4.587 | **0.0001** | 9914 |
|  | Transplant type (SE *vs.* DE) | 1 | 440.91 | 440.91 | 1.197 | 0.2391 | 9922 |
| Polar metabolites | Treatments  (CC, CA, AC, AA) | 3 | 5504.7 | 1834.9 | 2.0386 | **0.0002** | 9815 |
|  | Site of origin  (C *vs.* A) | 1 | 2891.7 | 2891.7 | 3.1087 | **0.0001** | 9883 |
|  | Transplant type (SE *vs.* DE) | 1 | 1595.1 | 1595.1 | 1.6306 | **0.0303** | 9888 |
| Proteins | Treatments  (CC, CA, AC, AA) | 3 | 416.83 | 138.94 | 2.3289 | **0.0277** | 7312 |
|  | Site of origin  (C *vs.* A) | 1 | 54.196 | 54.196 | 0.64526 | 0.628 | 462 |
|  | Transplant type (SE *vs.* DE) | 1 | 268.69 | 268.69 | 4.2961 | **0.0118** | 462 |

**Table S5.** Summary of haplotype identities based on COI data obtained from individuals of *Platynereis* spp. collected from either inside (high *p*CO_2_/low pH) or outside (low *p*CO_2_/high pH) the vented area. Haplotypes were compared with the *P.* c.f. *massiliensis* reference ecotype characterised and sequenced by Lucey et al. (2015) and the degree of genetic difference evaluated.

|  |  |  |  | **Comparison with *P.* c.f. *massiliensis* reference ecotype (Lucey et al., 2015)** | | |
| --- | --- | --- | --- | --- | --- | --- |
| **Haplotype** | **N** | **% collected inside vent** | **% collected outside vent** | **# pairwise differences** | **% pairwise differences** | **Identity matched (rank position)** |
| Platy-1 | 24 | 45.8 | 54.2 | 15 | 2.5 | 4 |
| Platy-2 | 12 | 33.3 | 66.6 | 19 | 3.1 | 8 |
| Platy-3 | 4 | 75 | 25 | 19 | 3.1 | 8 |
| Platy-4 | 1 | 0 | 100 | 20 | 3.3 | 9 |
| Platy-5 | 3 | 33.3 | 66.6 | 23 | 3.8 | 11 |
| Platy-6 | 1 | 0 | 100 | 21 | 3.5 | 10 |
| Platy-7 | 1 | 0 | 100 | 18 | 3.0 | 7 |
| Platy-8 | 1 | 0 | 100 | 17 | 2.8 | 6 |
| Platy-9 | 30 | 96.7 | 3.3 | 0 | 0 | 1 |
| Platy-10 | 1 | 0 | 100 | 18 | 3.0 | 7 |
| Platy-11 | 1 | 0 | 100 | 23 | 3.8 | 11 |
| Platy-12 | 1 | 0 | 100 | 17 | 2.8 | 6 |
| Platy-13 | 1 | 0 | 100 | 37 | 6.1 | 13 |
| Platy-14 | 1 | 0 | 100 | 24 | 4.0 | 12 |
| Platy-15 | 1 | 100 | 0 | 16 | 2.6 | 5 |
| Platy-16 | 1 | 100 | 0 | 7 | 1.2 | 2 |
| Platy-17 | 1 | 100 | 0 | 18 | 3.0 | 7 |
| Platy-18 | 1 | 100 | 0 | 14 | 2.3 | 3 |

**
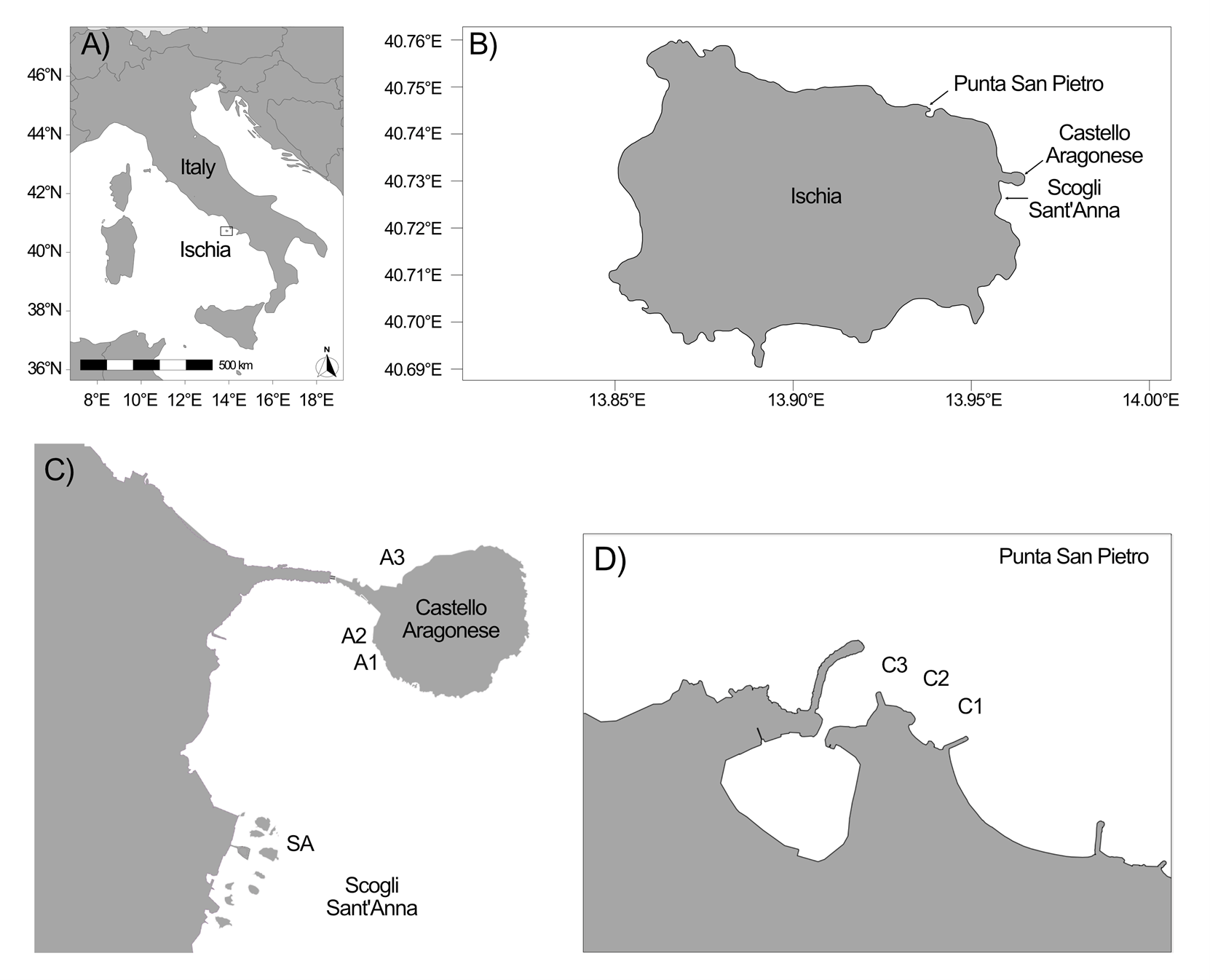
**

**Figure S1.** Map of the study area. **Box A:** the Island of Ischia in the Gulf of Naples, Italy. **Box B:** the Island of Ischia, with the Castello Aragonese, where the CO_2_ vents system is located. **Box C:** Castello Aragonese showing the area used for the high *p*CO_2_ collection site (stations A1 and A2) and deployment sites (stations A1, A2 and A3); the control (low *p*CO_2_) collection site (i) Scoglio Sant’Anna is also shown (SA). **Box D:** the site of Punta San Pietro showing the area used for the control collection site (ii) (stations C1, C2 and C3), which were also used as control (low *p*CO_2_) deployment site.


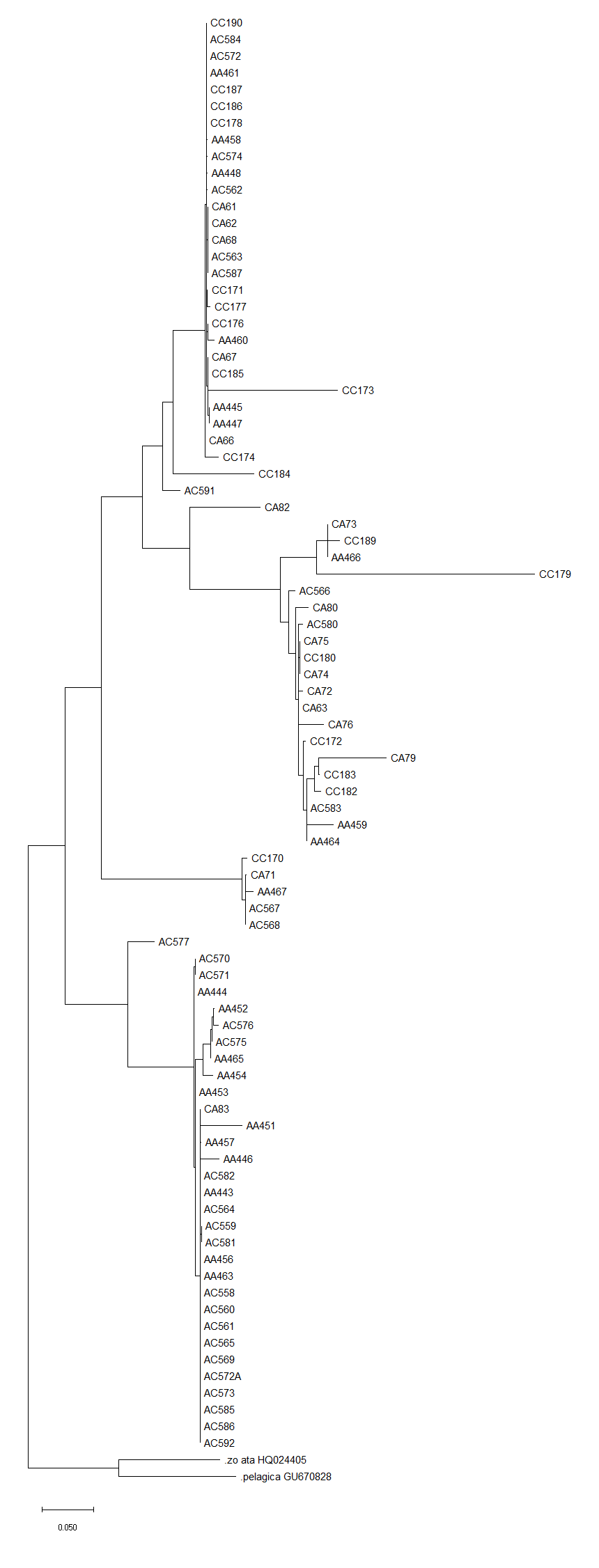


Platy-7

Platy-13

Platy-18

Platy-17

Platy-16

Platy-15

Platy-14

Platy-12

Platy-10

Platy-8

Platy-6

Platy-5

Platy-4

Platy-3

Platy-2

Platy-2

Platy-11

Platy-1

Platy-9

**b**

**a**

100

78

77

97

83

89

99

82

100

88

71

70

96

90

99

**Figure S2.** Maximum likelihood tree of Platynereis spp. COI sequence data. Branch support is indicated as bootstrap percentages (1,000 replicates); only values equal or greater to 70 are shown. Major clades designated by lower case letters. Haplotypes indicated by number e.g. Platy-1. White boxes = annelids collected in the low *p*CO_2_ area and grey boxes = annelids collected in high *p*CO_2_ area. Outgroup species are *Nereis zonata* and *Nereis pelagica*.

**
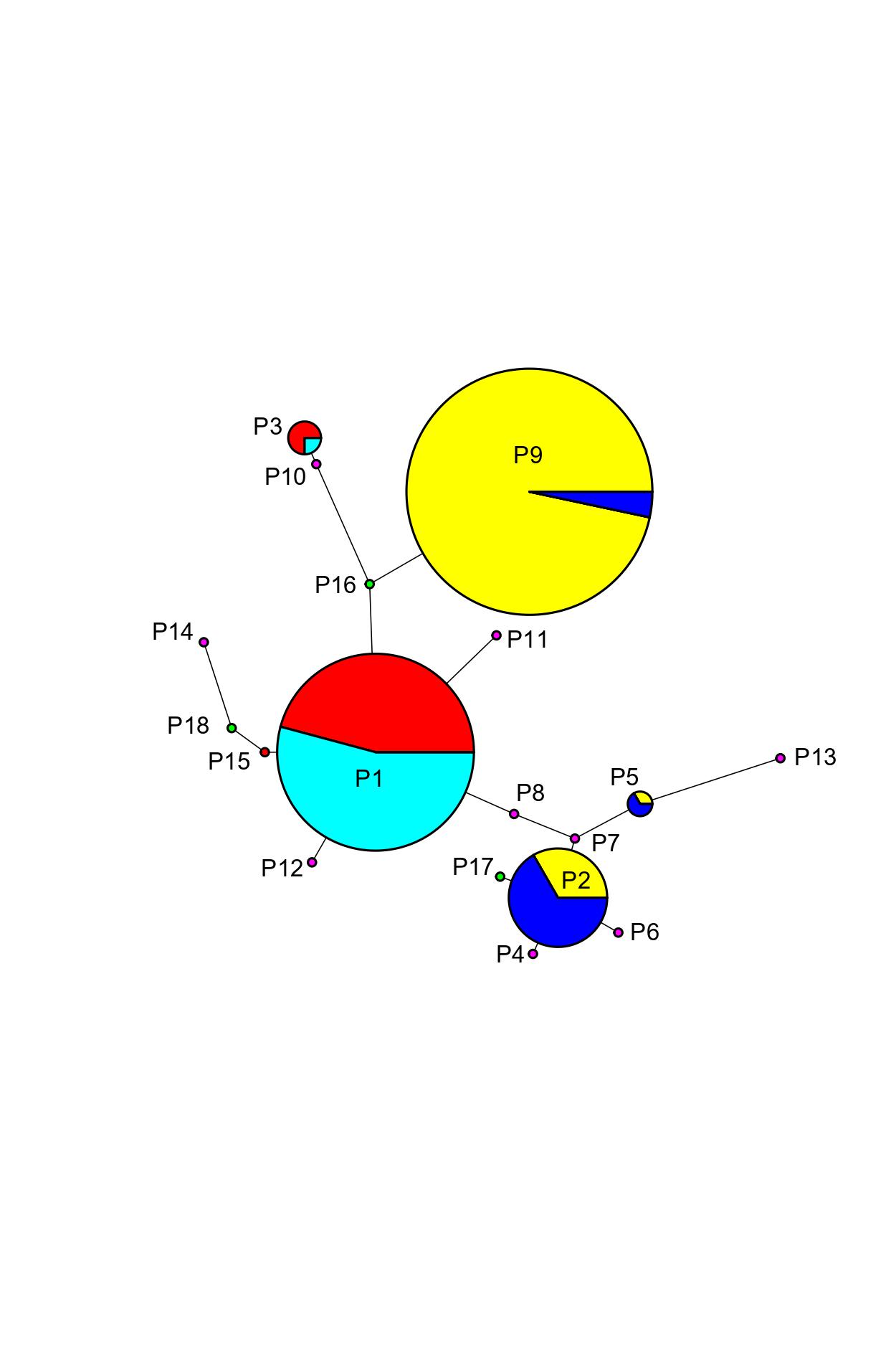
**

**Figure S3.** Haplotype network for 18 cytochrome oxidase I haplotypes (from 86 individuals) of *Platynereis* spp. (P1-18). Circles represent haplotypes, with the area of each circle proportional to the frequency with which it occurred in the whole sample set, and pie charts indicate the distribution of the haplotype between species and individuals collected inside or outside of the vented area. A preliminary haplotype analysis (Table S3) including all reference sequences as well as those generated during this study allowed the identification of haplotypes as either *P*. c.f. *massiliensis* or *P.* c.f. *dumerilii.* Unidentified haplotypes are designated *Platynereis* sp. Inside-vent collected *Platynereis* c.f. *massiliensis* = yellow, outside-vent collected *Platynereis* c.f. *massiliensis* = dark blue, inside-vent collected *Platynereis* c.f. *dumerilii* = red, outside-vent collected *Platynereis* c.f. *dumerilii* = light blue, inside-vent collected *Platynereis* sp. (green), and outside-vent collected *Platynereis* sp. (pink). Connecting lines show mutational pathways among haplotypes. Missing intermediates not shown.

**Table S10.** Enrichment analysis performed on the lipidome of *Platynereis* spp. according to site of origin (control versus acidified – CO2 vents). This analysis was based on a combination of mummichog and GSEA algorithms using the ‘MS Peaks to Pathways’ module in Metaboanalyst 4.0. Data was input as a table with m/z peaks, p-values and fold-changes. The following parameters were used in the analysis: molecular weight tolerance 5 ppm, negative mode, p-value <0.01 for peak significance in mummichog, database ‘lipids – main chemical class’, which contains 77 main lipid chemical class metabolite sets from RefMet. Significantly enriched lipid classes are highlighted in bold (p<0.05).

| **Pathway Name** | **Total** | **All Hits** | **Sig Hits** | **Mummichog p-value** | **GSEA p-value** | **Combined p-value** |
| --- | --- | --- | --- | --- | --- | --- |
| Hopanoids | 1 | 1 | 1 | 0.1751 | 0.05769 | 0.05653 |
| Glycerophosphoserines | 3031 | 1229 | 19 | 0.999 | **0.01136** | 0.06218 |
| Glycerophosphoinositols | 3219 | 783 | 8 | 0.999 | **0.0125** | 0.06722 |
| Diradylglycerols | 3114 | 863 | 38 | 0.999 | **0.01266** | 0.06792 |
| Glycosyldiradylglycerols | 3462 | 798 | 13 | 0.999 | **0.01316** | 0.07009 |
| Fatty esters | 569 | 90 | 6 | 0.9992 | **0.01408** | 0.07406 |
| Ceramides | 5424 | 2354 | 43 | 0.999 | **0.02273** | 0.1087 |
| Fatty acids | 799 | 255 | 4 | 0.999 | **0.04762** | 0.1924 |
| Sphingomyelins | 2315 | 997 | 9 | 0.999 | **0.04762** | 0.1924 |
| Monoradylglycerols | 73 | 31 | 2 | 0.9807 | 0.05263 | 0.2046 |
| Glycerophosphates | 2996 | 909 | 21 | 0.999 | 0.05556 | 0.216 |
| Phosphosphingolipids | 4581 | 1710 | 14 | 0.999 | 0.05556 | 0.216 |
| Glycerophosphocholines | 3120 | 1111 | 17 | 0.999 | 0.05882 | 0.2253 |
| Glycerophosphoethanolamines | 3153 | 1331 | 22 | 0.999 | 0.09091 | 0.3087 |
| Glycerophosphoglycerols | 3058 | 1049 | 13 | 0.999 | 0.09524 | 0.319 |
| Isoprenoids | 423 | 107 | 5 | 1.0 | 0.09589 | 0.3207 |
| Fatty amides | 141 | 37 | 3 | 0.9688 | 0.1111 | 0.3475 |
| Sterol esters | 86 | 10 | 1 | 0.8542 | 0.1556 | 0.4012 |
| Triradylglycerols | 27190 | 3913 | 30 | 0.999 | 0.2875 | 0.6455 |
| Steroids | 173 | 55 | 2 | 0.9997 | 0.375 | 0.7427 |
| Docosanoids | 43 | 27 | 1 | 0.9945 | 0.5614 | 0.8837 |
| Sphingoid bases | 78 | 20 | 1 | 0.9788 | 0.918 | 0.9947 |
| Sterols | 188 | 43 | 1 | 0.9998 | 0.9189 | 0.9966 |
| Glycosphingolipids | 13440 | 4877 | 28 | 0.999 | 0.9277 | 0.9972 |

**Table S11.** Differentially abundant proteins (*t*-tests with 250 randomizations, False Discovery Rate of 0.05 and s 0.1) between annelids *Platynereis* spp. reciprocally transplanted between the same environment (SE, including CC = collected in low *p*CO_2_/high pH and transplanted to low *p*CO_2_/high pH and AA = collected in high *p*CO_2_/low pH and transplanted to high *p*CO_2_/low pH) or different environments (DE, including CA = collected in low *p*CO_2_/high pH and transplanted to high *p*CO_2_/low pH, and AC =collected in high *p*CO_2_/low pH and transplanted to low *p*CO_2_/high pH). Only significant proteins are shown in the table.

| **- LOG (P-value)** | **Difference SE-DE** | **Protein ID (Uniprot accession_name)** |
| --- | --- | --- |
| 2.207398868 | -1.412004218 | P24733_Myosin heavy chain. striated muscle |
| 3.094215919 | -1.741633492 | Q8WZ42_Titin |
| 1.563042065 | -1.317128837 | Q7PPA5_Calcium-transporting ATPase sarcoplasmic endoplasmic reticulum type |
| 1.678432126 | -1.544133921 | Q5R546_ATP synthase subunit alpha, mitochondrial |
| 3.279476112 | -2.172819679 | P18238_ADP, ATP carrier protein 3 |
| 1.443736923 | -1.510957178 | B0BM40_Triosephosphate isomerase |
| 2.039860734 | -1.797083264 | Q00963_Spectrin beta chain |
| 1.350632397 | -1.313893666 | P24733_Myosin heavy chain, striated muscle |
| 4.683893095 | -2.265662491 | P24733_Myosin heavy chain, striated muscle |
| 2.487299438 | -1.824075687 | P13607_Sodium-potassium-transporting ATPase subunit alpha |
| 3.898601952 | -2.198435118 | P22700_Calcium-transporting ATPase sarcoplasmic endoplasmic reticulum type |
| 1.489343564 | -1.571587825 | O35115_Four and a half LIM domains protein 2 |
| 4.140460994 | -2.147077968 | P40926_Malate dehydrogenase, mitochondrial |
| 1.503162923 | -1.43530733 | Q8BTM8_Filamin-A |
| 1.492798397 | -1.538719182 | Q25145_Tropomyosin |
| 2.256865946 | -1.917924427 | Q5RFN6_Calponin-2 |
| 1.541463288 | -1.285915415 | P10719_ATP synthase subunit beta, mitochondrial |
| 2.865803623 | -1.674688342 | Q01240_60 kDa neurofilament protein |
| 3.004561698 | -1.577265655 | P51546_Glycocyamine kinase |
| 2.054575295 | -1.371036103 | O96064_Paramyosin |
| 2.275367161 | -1.785275087 | P13578_Extracellular globin-2B |
| 1.370880601 | -1.431423346 | Q4R502_Isocitrate dehydrogenase [NADP], mitochondrial |
| 1.826582395 | -1.555404648 | Q9UBQ7_Glyoxylate reductase/hydroxypyruvate reductase |
| 4.588762251 | -2.209651972 | Q0VCX2_78 kDa glucose-regulated protein |
| 1.935888643 | -1.696788947 | Q8BTM8_Filamin-A |

**Table S12:** Summary of the omics analyses carried out on each individual *Platynereis* sp.

| **Treatment** | **Station** | **Chamber** | **Sample ID** | **Omics analysis** |
| --- | --- | --- | --- | --- |
| CC | C1 | A | 171 | metabolomics |
| CC | C1 | A | 172 | metabolomics |
| CC | C1 | A | 174 | metabolomics |
| CC | C1 | A | 176 | proteomics |
| CC | C2 | A | 177 | proteomics |
| CC | C2 | A | 181 | proteomics |
| CC | C2 | A | 182 | proteomics |
| CC | C2 | A | 183 | proteomics |
| CC | C3 | A | 185 | metabolomics |
| CC | C3 | A | 186 | metabolomics |
| CC | C3 | A | 187 | metabolomics |
| CC | C3 | A | 188 | metabolomics |
| CC | C1 | B | 189 | metabolomics |
| CC | C1 | B | 190 | proteomics |
| CA | A1 | A | 61 | metabolomics |
| CA | A1 | A | 62 | metabolomics |
| CA | A1 | A | 63 | proteomics |
| CA | A2 | A | 66 | metabolomics |
| CA | A2 | A | 67 | metabolomics |
| CA | A2 | A | 68 | proteomics |
| CA | A2 | A | 69 | proteomics |
| CA | A3 | A | 71 | metabolomics |
| CA | A3 | A | 73 | metabolomics |
| CA | A3 | A | 74 | metabolomics |
| CA | A3 | A | 77 | proteomics |
| CA | A3 | A | 79 | proteomics |
| CA | A3 | A | 82 | metabolomics |
| CA | A1 | B | 83 | proteomics |
| CA | A1 | B | 85 | proteomics |
| AC | C1 | A | 558 | metabolomics |
| AC | C1 | A | 562 | proteomics |
| AC | C1 | A | 564 | proteomics |
| AC | C1 | A | 567 | proteomics |
| AC | C1 | B | 570 | metabolomics |
| AC | C1 | B | 573 | metabolomics |
| AC | C1 | B | 574 | metabolomics |
| AC | C1 | B | 575 | metabolomics |
| AC | C1 | B | 576 | metabolomics |
| AC | C1 | B | 579 | proteomics |
| AC | C1 | B | 580 | proteomics |
| AC | C2 | A | 582 | proteomics |
| AC | C2 | A | 586 | metabolomics |
| AC | C2 | A | 588 | proteomics |
| AC | C3 | A | 592 | metabolomics |
| AA | A1 | B | 443 | proteomics |
| AA | A1 | B | 445 | metabolomics |
| AA | A1 | B | 446 | metabolomics |
| AA | A1 | B | 448 | metabolomics |
| AA | A1 | B | 451 | proteomics |
| AA | A2 | A | 452 | metabolomics |
| AA | A2 | A | 453 | metabolomics |
| AA | A2 | A | 454 | metabolomics |
| AA | A2 | A | 455 | proteomics |
| AA | A2 | A | 456 | proteomics |
| AA | A2 | A | 458 | proteomics |
| AA | A2 | A | 459 | proteomics |
| AA | A3 | A | 462 | metabolomics |
| AA | A3 | A | 465 | metabolomics |
| AA | A3 | A | 467 | proteomics |

**References**

Chong, J., Wishart, D.S., and Xia, J. (2019). Using MetaboAnalyst 4.0 for comprehensive and integrative metabolomics data analysis. Curr. Protoc. Bioinforma. 68: 1–128. doi: org/10.1002/cpbi.86

Conzelmann, M., Williams, E.A., Krug, K., Franz-Wachtel, M., Macek, B., and Jékely G. (2013). The neuropeptide complement of the marine annelid Platynereis dumerilii. BMC Genomics 14:906. doi: 10.1186/1471-2164-14-906

Cox, J., and Mann, M. (2008). MaxQuant enables high peptide identification rates, individualized p.p.b.-range mass accuracies and proteome-wide protein quantification. Nat. Biotechnol. 26: 1367-1372. doi: 10.1038/nbt.1511

Dickson, A.G. (1990). Thermodynamics of the dissociation of boric acid in synthetic seawater from 273.15 to 318.15 K. Deep. Sea. Res. 37, 755-766.

Dickson, A.G., and Millero, F.J. (1987). A comparison of the equilibrium constants for the dissociation of carbonic acid in seawater media. Deep Sea. Res. 34, 1733-1743.

Excoffier, L., Laval, G., and Schneider, S. (2005). Arlequin ver. 3.0: an integrated software package for population genetics data analysis. Evol. Bioinformatics Online 1: 47–50.

Fahy, E., Sud, M., Cotter, D., and Subramaniam, S. (2007). LIPID MAPS online tools for lipid research. Nucleic Acids Res. 35:W606–W612. doi: 10.1093/nar/gkm324

Folmer, O., Black, M., Hoen, W., Lutz, R., and Vrijenhoek, R. (1994). DNA primers for amplification of mitochondrial cytochrome subunit I from diverse metazoan invertebrates. Mol. Mar. Biol. Biotech. 3, 294–299.

Hall, T.A. (1999). BioEdit: a user-friendly biological sequence alignment editor and analysis program for Windows 95/98/NT. Nucleic Acids Symp. Ser. 41, 95–98.

Kirwan, J. A., Weber, R.J.M., Broadhurst, D.I., and Viant, M.R. (2014). Direct infusion mass spectrometry metabolomics dataset: a benchmark for data processing and quality control. Sci. Data. 1:140012. doi: 10.1038/sdata.2014.12

Kanehisa, M., Goto, S., Sato, Y., Furumichi, M., and Tanabe, M. (2012). KEGG for integration and interpretation of large-scale molecular data sets. Nucleic Acids Res. 40, D109–D114. doi: 10.1093/nar/gkr988

Kanehisa, M., Furumichi, M., Sato, Y., Matsuura, Y. and Ishiguro-Watanabe, M. (2025). KEGG: biological systems database as a model of the real world. Nucleic Acids Res. 53, D672-D677. doi: 10.1093/nar/gkae909

Kumar, S., Stecher, G., Li, M., Knyaz, C., and Tamura, K. (2018). MEGA X: Molecular Evolutionary Genetics Analysis across computing platforms. Mol. Biol. Evol. 35:1547-1549. doi: 10.1093/molbev/msy096

Li, S., Park, Y., Duraisingham, S., Strobel, F.H., Khan, N., Soltow, Q.A., et al. (2013). Predicting network activity from high throughput metabolomics. PLoS. Comput. Biol. 9:e1003123. doi: 10.1371/journal.pcbi.1003123

Lucey, N., Lombardi, C., DeMarchi, L., Schulze, A., Gambi, M.C., and Calosi, P. (2015). To brood or not to brood: Are marine invertebrates that protect their offspring more resilient to ocean acidification? Sci. Rep. 5:12009. doi: 10.1038/srep12009

Mehrbach, C., Culberson, C.H., Hawley, J.E., and Pytkowicz, R.M. (1973). Measurement of the apparent dissociation constants of carbonic acid in seawater at atmospheric pressure. Limnol. Oceanogr. 18, 897-907.

Paradis E. (2010). pegas: an R package for population genetics with an integrated-modular approach. Bioinformatics, 26:419-420. doi: 10.1093/bioinformatics/btp696

Pierrot, D., Lewis, E., and Wallace, D.W.R. (2006). MS Excel program developed for CO_2_ system calculations, ORNL/CDIAC-105. Oak Ridge, TN: Carbon Dioxide Information Analysis Center, Oak Ridge National Laboratory, U.S. Department of Energy.

Posada, D., and Crandall, K.A. (1998). MODELTEST: testing the model of DNA substitution. Bioinformatics, 14:817–818.

Rozas, J., Ferrer-Mata, A., Sanchez-DelBarrio, J.C., Guirao-Rico, S., Librado, P., Ramos-Onsins, S.E., et al. (2017). DnaSP 6: DNA sequence polymorphism analysis of large data sets. Mol. Biol. Evol. 34:3299-3302. doi: 10.1093/molbev/msx248

Sokolov, E.P. (2000). An improved method for DNA isolation from mucopolysaccharide rich molluscan tissues. J. Molluscan Stud. 66, 573–575.

Templeton A.R., Crandall K.A., and Sing C.F. (1992). A cladistic analysis of phenotypic associations with haplotypes inferred from restriction endonuclease mapping and DNA sequence data. III. Cladogram estimation. Genetics. 132, 619–633.

Tyanova, S., Temu, T., Sinitcyn, P., Carlson, A., Hein, M.Y., Geiger, T., et al. (2016). The Perseus computational platform for comprehensive analysis of (prote)omics data. Nat. Methods 13:731-740. doi: 10.1038/nmeth.3901

Weber, R.J.M., and Viant, M.R. (2010). MI-Pack: increased confidence of metabolite identification in mass spectra by integrating accurate masses and metabolic pathways. Chemometr. Intell. Lab. 104, 75–82. doi: 10.1016/j.chemolab.2010.04.010

Wenk, M.R. (2019). Encyclopedia of Lipidomics. Dordrecht: Springer. doi: 10.1007/978-94-007-7864-1
